# Intracellular and intercellular transport of RNA organelles in CXG repeat disorders: The strength of weak ties

**DOI:** 10.3389/fmolb.2022.1000932

**Published:** 2022-12-16

**Authors:** Deepti Kailash Nabariya, Annika Heinz, Sabrina Derksen, Sybille Krauß

**Affiliations:** Human Biology/Neurobiology, Institute of Biology, Faculty IV, School of Science and Technology, University of Siegen, Siegen, Germany

**Keywords:** RNA granules, processing bodies (p-bodies; PB), stress granule (SG), exosomes, CXG repeat disorders, RNA toxicity, neurodegeneration, neuronal RNA granules

## Abstract

RNA is a vital biomolecule, the function of which is tightly spatiotemporally regulated. RNA organelles are biological structures that either membrane-less or surrounded by membrane. They are produced by the all the cells and indulge in vital cellular mechanisms. They include the intracellular RNA granules and the extracellular exosomes. RNA granules play an essential role in intracellular regulation of RNA localization, stability and translation. Aberrant regulation of RNA is connected to disease development. For example, in microsatellite diseases such as CXG repeat expansion disorders, the mutant CXG repeat RNA’s localization and function are affected. RNA is not only transported intracellularly but can also be transported between cells *via* exosomes. The loading of the exosomes is regulated by RNA-protein complexes, and recent studies show that cytosolic RNA granules and exosomes share common content. Intracellular RNA granules and exosome loading may therefore be related. Exosomes can also transfer pathogenic molecules of CXG diseases from cell to cell, thereby driving disease progression. Both intracellular RNA granules and extracellular RNA vesicles may serve as a source for diagnostic and treatment strategies. In therapeutic approaches, pharmaceutical agents may be loaded into exosomes which then transport them to the desired cells/tissues. This is a promising target specific treatment strategy with few side effects. With respect to diagnostics, disease-specific content of exosomes, e.g., RNA-signatures, can serve as attractive biomarker of central nervous system diseases detecting early physiological disturbances, even before symptoms of neurodegeneration appear and irreparable damage to the nervous system occurs. In this review, we summarize the known function of cytoplasmic RNA granules and extracellular vesicles, as well as their role and dysfunction in CXG repeat expansion disorders. We also provide a summary of established protocols for the isolation and characterization of both cytoplasmic and extracellular RNA organelles.

## 1 Introduction

RNA-protein interactions are of prime importance in many cellular processes, both in healthy as well as disease conditions. Proteins recognize specific structural elements of RNA with RNA-hairpin structures being an important example. Abnormal hairpin structures aberrantly recruit cellular proteins, for example in monogenic diseases caused by expanded CXG repeat hairpin-motifs. CXG repeat diseases are a group of neurodegenerative diseases caused by a pathogenic expansion of a CXG repeat in the disease-causing genes (reviewed in (Orr and Zoghbi, 2007)) (Table 1). They include, for example, myotonic dystrophy type 1 (DM1), Huntington’s disease (HD), fragile X syndrome (FXS), and several of the spinocerebellar ataxias (SCAs). In CXG repeat diseases, the mutant repeat can be located either in the coding or in the non-coding region of the respective gene (Table 1). If the repeat lies within the coding region, a protein is translated that contains an expanded stretch of the repeat-encoded amino acid. For example, pathogenic CAG repeats translate into a poly-glutamine stretch, or CGG repeats encode a poly-arginine stretch. The resulting mutant proteins may act toxic to cells by a pathogenic gain of function and often tend to aggregate. A prominent example is the mutant Huntingtin (HTT) protein, which is aggregation -prone and has been linked to neurotoxicity in HD. The general mode of abnormal function of mutant proteins like HTT is linked to aberrant binding and sequestration of diverse proteins into aggregates, which affects their physiological function (reviewed in (Arrasate and Finkbeiner, 2012)). Besides this, RNA-dependent toxicity is also relevant. In contrast to physiological RNAs with short CXG repeats, the expanded CXG repeats within mutant RNAs fold into characteristic hairpin structures (reviewed in (Thornton, 2006)). These pathogenic hairpins trap various proteins, thereby impeding their function (reviewed in (Nalavade et al., 2013; Griesche et al., 2016; Schilling et al., 2019; Heinz et al., 2021a; Heinz et al., 2021b; Heinz et al., 2021c). For example, several of these proteins belong to the splicing machinery. Consequently, splicing is affected (Paul et al., 2011; Sathasivam et al., 2013; Schilling et al., 2019). The importance of RNA-mediated toxicity is highlighted by the fact that a neurodegenerative phenotype develops, even if the CXG repeat lies in the untranslated region. To date, there is no cure for CXG repeat expansion diseases and the available treatments are of palliative nature only. This indicates the need for a better understanding of molecular pathomechanisms and, based on this, the development of innovative therapeutic approaches.

**TABLE 1:** CXG repeat diseases.

Disease	Gene	Expanded CXG repeat	Non-pathogen number of repeats	Pathogenic number of repeats	Repeat in coding or non-coding	References
Huntington’s disease	*HTT*	CAG	9–36	≥40	Polyglutamine protein	Myers (2004)
Spinocerebellar ataxia type 1	*ATXN1*	CAG	19–36	43–81	Polyglutamine protein	Chung et al. (1993)
Spinocerebellar ataxia type 2	*ATXN2*	CAG	13–31	32–79	Polyglutamine protein	Almaguer-Mederos et al. (2010)
Spinocerebellar ataxia type 3	*ATXN3*	CAG	13–36	68–79	Polyglutamine protein	Kawaguchi et al. (1994)
Spinocerebellar ataxia type 6	*CACNA1A*	CAG	4–18	19–33	Polyglutamine protein	Li et al. (2009)
Spinocerebellar ataxia type 7	*ATXN7*	CAG	4–37	37- ca. 200	Polyglutamine protein	Stevanin et al. (1998)
Spinocerebellar ataxia type 17	*TBP*	CAG	21–42	37–48	Polyglutamine protein	Hire et al. (2011)
Dentatorubropallidoluysian atrophy	*ATN1*	CAG	7–23	49–75	Polyglutamine protein	Koide et al. (1994)
Spinal and bulbar muscular atrophy	*AR*	CAG	9–36	38–62	Polyglutamine protein	Fratta et al. (2014)
Spinocerebellar ataxia type 12	*PPP2R2B*	CAG	7–28	55–78	Non-coding	O'Hearn et al. (2012)
Huntington disease like-2	*JPH3*	CAG/CTG	6–28	>41	Non-coding	Todd and Paulson, (2010)
Spinocerebellar ataxia type 8	*ATXN8*	CAG	15–50	71–1,300	CAG: Polyglutamine protein	Ikeda et al. (2000)
	*ATXN8OS* (opposite strand of *ATXN8*)	CTG				
					CTG: Non-coding	
Myotonic dystrophy Type 1	*DMPK*	CTG	-	>50	Non-coding, 3′UTR	Musova et al. (2009)
Fragile X syndrome	*FMR1*	CGG	Up to 55	>200	Non-coding, 5′UTR	(reviewed in (Willemsen et al., 2011))
						LaCroix et al. (2019)
Baratela-Scott syndrome	*XYLT1*	GGC	9–20	-	Non-coding, 5′UTR	Al-Mahdawi et al. (2008)
Friedreich’s ataxia	*FXN*	GAA	5–30	70- more than 1,000	Non-coding, intron	Matheny et al. (2019)

^a^ Source: OMIM. https://www.omim.org/.

Recent studies suggest that the subcellular localisation of mutant RNA is an essential aspect of disease development. From the moment an RNA is transcribed, a complex cascade of associations with various RNA-binding proteins to form ribonucleoprotein complexes that accomplish precise regulatory function, is initiated. RNA-binding proteins determine the functional fate of RNA (reviewed in (Schoenberg and Maquat, 2012)). Often, these ribonucleoprotein complexes cluster to form spherical, microscopically visible, large membrane-less compartments called RNA granules (reviewed in (Anderson and Kedersha, 2006; Anderson and Kedersha, 2009; Buchan and Parker, 2009; Fan and Leung, 2016; An et al., 2021)). RNA granules control various steps of RNA metabolism, including translation, mRNA trafficking, splicing, silencing and RNA decay (reviewed in (Decker and Parker, 2012)). They are exceptionally dynamic and demonstrate functional plasticity. While each kind of RNA granule has its own signature of proteins and transcripts, different types of granules also share a subset of proteins and RNAs, which helps forming a cross-linked collaborative system of RNA granules. This network basically adjusts the destination of a particular biomolecule to facilitate and support various cellular functions, be it stress, transport, development, or decay (reviewed in (An et al., 2021)). Due to this extensive cross-talk, there is a frequent exchange of macromolecules between granules when in close proximity (reviewed in (Decker and Parker, 2012)). Moreover, the lack of a membrane barrier eases this exchange of biomolecules from one granule to another or their neighbouring cellular components and *vice versa* (reviewed in (KD, 2017)). Therefore, one can understand RNA granules better as a network and not as independent entities.

**FIGURE 1:**
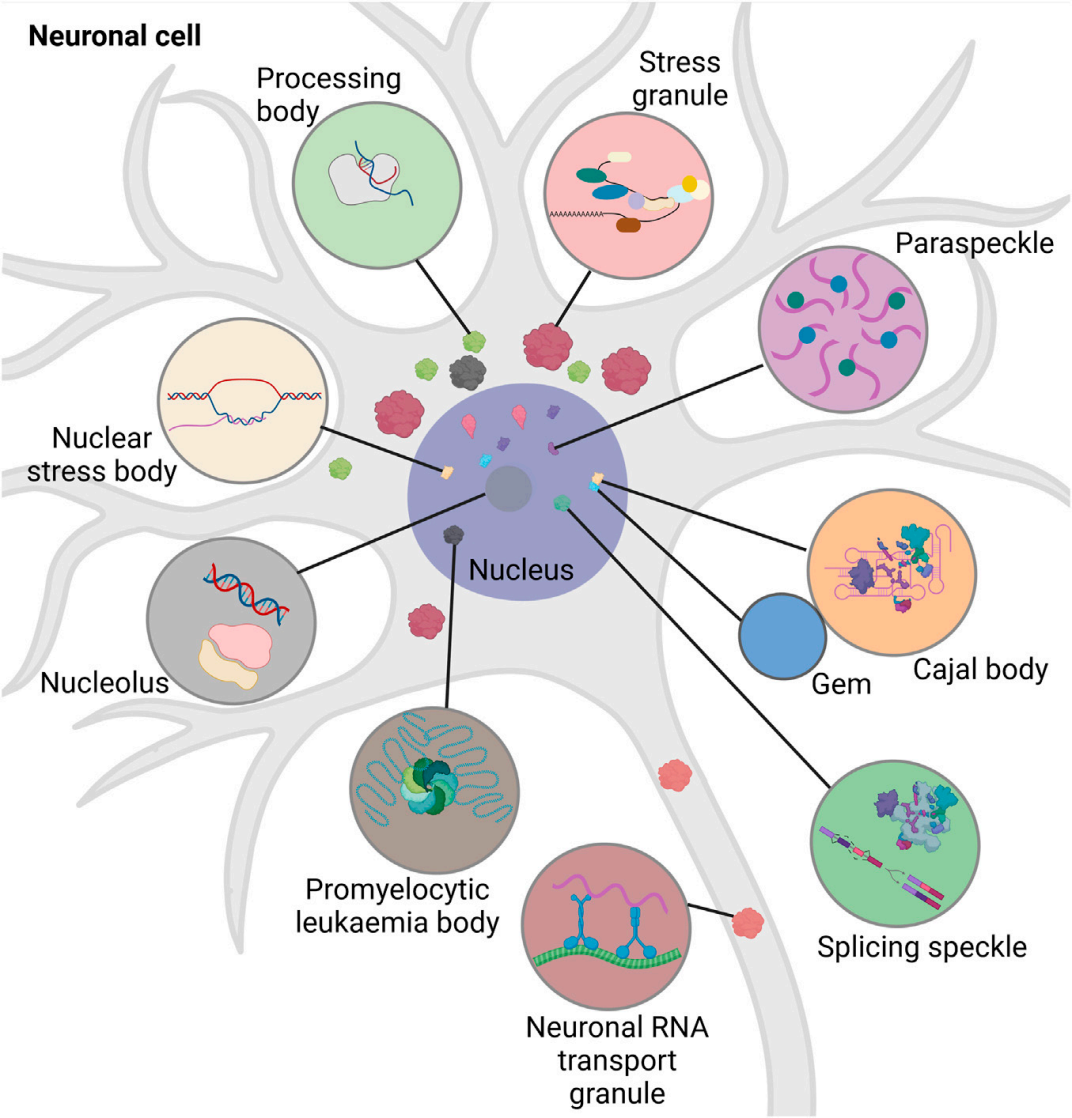
Schematic overview of different types of intracellular RNA granules in neuronal cell. Promyelocytic leukemia bodies belong to the nuclear granules. Nuclear granules (nucleus dark purple) include nuclear stress bodies (beige, sites of reprogramming of gene expression), Cajal bodies (orange, sites of RNA-processing), Gems (blue, Cajal body assistance), splicing speckles (green, sites of splicing factor modification and storage), paraspeckles (purple, sites of RNA-retention) and the nucleolus (dark grey, site of ribosomal synthesis). Cytoplasmic granules include stress granules (red, sites of translational block), neuronal RNA transport granules (brown, sites of neuronal RNA-transport), and processing bodies (light green, sites of RNA-storage and -decay). Created with BioRender.com.

Intracellular RNA granules differ with respect to their localization, composition, and size. Based on their localization, they are mainly divided into two groups: Nuclear and cytoplasmic (Figure 1). Nuclear granules include nucleoli, splicing speckles, paraspeckles, promyelocytic leukaemia bodies, nuclear stress bodies, Cajal bodies and Gems (Gemini of the Cajal bodies) (reviewed in (An et al., 2021; Biamonti and Vourc’h, 2010; Fox and Lamond, 2010; LL, 2019; Decker et al., 2022) (Moujaber and Stochaj, 2018)).

Nuclear granules are integral for regulating cell growth, proliferation, gene expression, splicing, and survival. The nucleoli help in ribosomal assembly, contribute to DNA repair, modulate stress-response, apoptosis, and senescence (reviewed in (Tsekrekou et al., 2017)). Cajal bodies play an important role in ribosome and telomerase biogenesis, recycling of the components involved in splicing, and assembly of snRNPs (reviewed in (DS, 2017)). The large irregular speckles are the storerooms for splicing factors and their modification. They attach to regulatory epigenetic proteins and help in chromatin organization, DNA repair, and RNA modification (reviewed in (Tripathi et al., 2010; Galganski et al., 2017)). While the paraspeckles modulate the processing of pri-miRNA and regulate gene expression by retaining transcription factors, the promyelocytic leukaemia bodies are involved in DNA replication and damage repair, quality control of nuclear proteins, oxidative stress response and telomere lengthening (reviewed in (Fox et al., 2018; Lallemand-Breitenbach and de Thé, 2018)). Nuclear stress bodies form in response to heat shock and are the place for phosphorylation of splicing factors (reviewed in (20)). Although separated by a nuclear envelope, RNA granules of the nucleus and the cytoplasm exchange proteins and communicate regularly (Khanna et al., 2014; Mahboubi and Stochaj, 2014). Nuclear granules have been extensively reviewed elsewhere (An et al., 2021; Biamonti and Vourc’h, 2010; Fox and Lamond, 2010; LL, 2019; Decker et al., 2022). Cytoplasmic RNA granules are categorized into stress granules (SGs), processing bodies (p-bodies, PBs), and neuronal RNA transport granules. The distribution of the cytoplasmic granules is dependent on their interaction with the cytoskeleton. They often associate with microtubules to promote their disassembly, mobilization, fusion and exchange of biomolecules (reviewed in (Moujaber and Stochaj, 2018)). Thus, cytoplasmic RNA granules play an important role in intracellular RNA transport and signalling.

Besides the intracellular RNA transport that is crucial to fulfil tightly spatiotemporally regulated RNA-function, RNA can also be transported intercellularly, thus contributing to cell-cell communication. For this intercellular transport, extracellular vesicles (EVs) play an essential role.

In this review, our focus will be on function and dysfunction of cytoplasmic and extracellular RNA organelles in CXG repeat expansion diseases.

## 2 Cytoplasmic RNA granules

### 2.1 Stress granules

**FIGURE 2:**
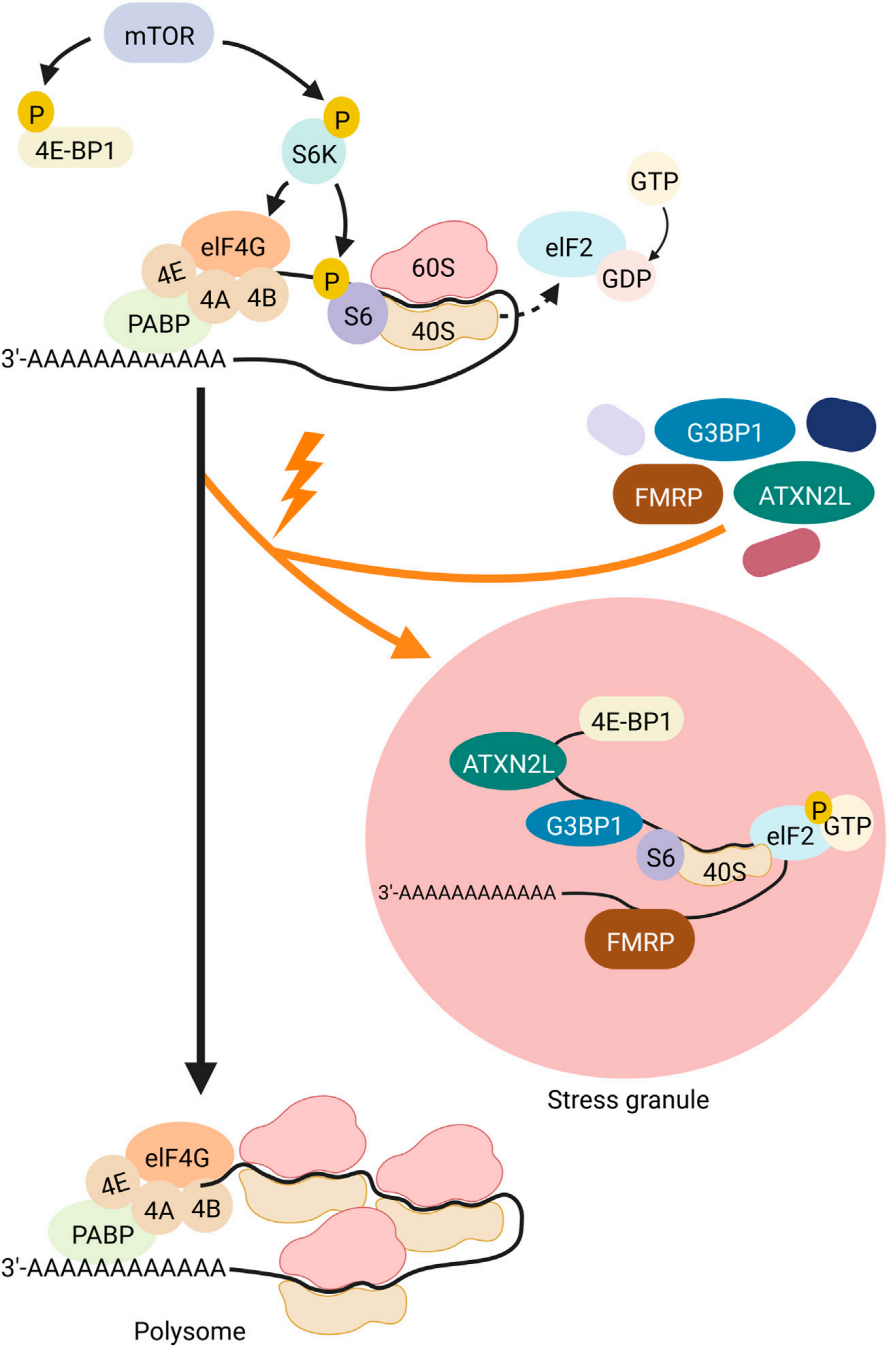
Schematic representation of translational induction and translational block *via* SG-formation. The kinase mTOR (gray) controls the phospho-dependent activity of S6K (purple) and 4E-BP1 (yellow). 4E-BP1 is a negative regulator of translation that represses translation when attached to the 5′ end of an mRNA. Its phosphorylation by mTOR leads to its detachment from the RNA and the release of this translational block. Simultaneously, S6K is phospho-activated by mTOR. Activated S6K phosphorylates its target S6 (purple), a ribosomal subunit. These two mTOR-dependent phosphorylation events promote assembly of the ribosome at the RNA and thus translation. Hydrolysis of GTP to GDP at the protein elF2 (light blue) releases the binding of elF2 to the 40S subunit of the ribosome, allowing the ribosome to assemble and subsequently polysome formation. Upon cell stress (indicated by an orange flash), elF2 is phosphorylated, thus cannot detach from the 40S unit, thereby blocking translation and inducing polysome disassembly. Furthermore, 4E-BP1 is dephosphorylated and thus sticks to the 5′end of the mRNA also inhibiting translation. In addition, proteins such as FMRP (brown), ATXN2L (green) and G3BP1 (dark blue) organize the stalled RNA into SGs. Created with BioRender.com.

SGs are one type of RNA granules that can assemble in the cytosol. SGs are approximately 100–200 nm in size (reviewed in (SDL, 2006; Moujaber and Stochaj, 2018; An et al., 2021)). Environmental stresses such as viral infections, oxidative stress, ultraviolet (UV) radiation, and hypoxia are causative for the formation of SGs, as is heat, which led to the first description of SGs in cells (reviewed in (Anderson and Kedersha, 2009)). Immunostaining analyses identified a large number of proteins and RNAs as SG components. Many of them are preinitiation and translation-related factors, suggesting that SGs regulate translation induction (reviewed in (Anderson and Kedersha, 2009)). Translation induction is controlled by the mTOR (Mechanistic Target Of Rapamycin) signaling cascade (Figure 2). In this pathway, mTOR phosphorylates its two target proteins 4E-BP1 (eukaryotic translation initiation factor 4E-binding protein 1) and S6K (p70S6 kinase). One hand, the negative translation regulator 4E-BP1, that silences translation when attached to the 5′ end of an mRNA, is inactivated by phosphorylation *via* mTOR. As a result, 4E-BP1 unleashes the RNA and loses binding to elF4E (eukaryotic translation initiation factor 4E). ElF4E can then bind to elF4G and eIF4A, and the resulting complex binds to the preinitiation complex and the 5′UTR of the RNA. On the other hand, phospho-activated S6K phosphorylates and activates the ribosomal subunit S6. The preinitiation complex includes the small ribosomal subunit, the methionine-loaded start tRNA, the initiation factor eIF2, and GTP (guanosine triphosphate). GTP hydrolysis then releases eIF2 so that the large and small ribosomal subunits can complete forming the 80S ribosome.

Different types of environmental stresses activate different kinases that phosphorylate eIF2. Phosphorylation of eIF2a reduces the formation of the ternary complex and thus prevents the assembly of the preinitiation complex. This inhibits translation induction, which in turn leads to polysome degradation. Transcripts that are no longer polysome-bound, are then actively organised into SGs through RNA-binding proteins), which oligomerize RNA (Figure 2). Examples of such RNA-binding proteins are TIA-1/TIAR (TIA1 Cytotoxic Granule Associated RNA Binding Protein), FMRP (Fragile X Mental Retardation Protein), G3BP (ras-gap SH3-binding protein), CPEB (cytoplasmic polyadenylation binding protein), SMN (Survival of Motor Neurons protein), smaug, and TTP (tristetraprolin). Overexpression of any of these proteins trigger SG assembly even in the absence of stress. TIA-1 and CPEB have prion-like aggregation properties, while G3BP has an oligomerisation domain that is regulated by phosphorylation. Self-aggregation of SMN appears to be mediated by its YG-box domain, while aggregation of FMRP family members sems to rely on the coiled-coil domain. The redundancy of these RNA-protein interactions suggests that the formation of SGs can occur under different conditions through different interactions. For example, during oxidative stress in mammalian cells, the G3BP1 and G3BP2 paralogues play important roles in the formation of SGs through both self-interaction (Souquere et al., 2009) and interaction with the RNA-binding protein CAPRIN (Cytoplasmic Activation- And Proliferation-Associated Protein) (Jain et al., 2016). However, G3BP1/2 and CAPRIN are not required for SG assembly in response to osmotic stress (Tourrière et al., 2003). During glucose stress in yeast, Gtr1, Rps1b, and Hgh1 promote SG formation but suppress it during heat shock. Therefore, the mechanism of SG formation may be stress-specific (Solomon et al., 2007). This suggests that SGs may assemble differently in response to specific cellular conditions and have different functions in different stress situations.

However, in some health-relevant situations, the SG formation is changed. For example, during viral infections, viral proteins, such as the influenza A virus protein NS1 (non-structural protein 1), inhibit the formation of SGs. Encephalomyocarditis virus (EMCV) proteins, as well as poliovirus proteins also inhibit SG formation *via* G3BP cleavage (White et al., 2007). One reason for this viral mechanism may be that SGs link cellular stress responses to immune system activation. G3BP-induced SGs recruit and activate numerous antiviral proteins like PKR (RNA-dependent protein kinase). PKR induces the transcription factor NF-κB (nuclear factor kappa B), thereby triggering an immune response *via* interferons (IFN). In addition, viral RNA and antiviral proteins such as retinoic acid-inducible gene I-like receptors (RLRs) can localize into SGs, thereby changing the normal SG composition. RLRs detect cytoplasmic viral RNA and trigger the production of IFNs (Onomoto et al., 2012; Yang et al., 2014; Kedersha et al., 2016). These data provide solid evidence for a close link between antiviral response by the immune system and SG formation and also highlights the importance of SGs in biological cell responses.

Another way in which SGs can influence cellular processes is by sequestering and thereby limiting the access to cytosolic proteins. SGs modulate signaling cascades, for example, by recruiting components of the mTOR, RACK1 (Receptor For Activated C Kinase 1) or TRAF2 (TNF Receptor Associated Factor 2) signaling pathways (Reineke et al., 2015; Reineke and Lloyd, 2015). Since SGs additionally sequester numerous proteins involved in RNA physiology and metabolism, the formation of SGs likely has far-reaching effects on cell physiology. However, it is not yet fully understood how SG formation affects cell regulation and function, and future studies are needed to address this.

### 2.2 Processing bodies

Another group of cytoplasmic RNA granule is the PBs which are approximately ≥100 nm in size (reviewed in (24)). PBs consist mainly of mRNAs in conjunction with proteins responsible for translational repression and 5′-to-3′-mRNA decay (reviewed in (Arimoto et al., 2008; Takahara and Maeda, 2012). They were first discovered in 1997 when XRN1, a cytoplasmic 5′→3′ exoribonuclease, was observed in eukaryotic cells in granular structures (reviewed in (Parker and Sheth, 2007)). Subsequent studies from 2002 onwards addressed their possible modes of function (reviewed in (Decker and Parker, 2012)). PBs share similarities with other RNA granules, such as Cajal bodies, nucleoli, and SGs. For example, PBs and SGs share some common proteins, they inter-arrange, and formation of both can be triggered by cellular stress. Despite these similarities, PBs differ widely from other RNA granules in their molecular composition and function. RNA decay, for which PBs are mainly known, begins with decapping of the mRNA by the decapping enzyme complex, followed by 5′-to-3′ degradation by the exonuclease XRN1 (reviewed in (Arimoto et al., 2008)).

Contrary to the initial assumption that PBs are pure sites of 5′-to-3′ degradation of RNA, they also contain factors involved in mRNA regulation, RNA interference and translational suppression, although it should be noted that the formation of PBs is not essential for all these processes (Eulalio et al., 2007). Upon translocation into the PBs, mRNAs can be temporarily stored in a repressed state to be later released from the PBs and undergo translation in the cytoplasm. In some cell types, for example neurons that have long neurites, it is essential to have a local control of RNA stability and translation. Thus, PBs represent an important tool to locally degrade or store translationally repressed RNA in neurites. However, it is not known yet, if PBs are assembled locally inside the neurites, or if they are assembled in the soma and then translocated from the soma to synapses (Zeitelhofer et al., 2008).

Several factors influence and modulate PB formation. Just as in SG assembly, polysome-disassembly is relevant for PB assembly (Eulalio et al., 2007). In addition, decapping coactivators are responsible for PB formation (Coller and Parker, 2005). Furthermore, post-translational protein modifications play an important role in regulating PB assembly. For example, many PB proteins are phosphorylated (Yoon et al., 2010). Moreover, ubiquitination also plays a role in modulating PB formation. For example, Dcp1A is both mono- and polyubiquitinated at many of its lysine residues. This polyubiquitination of Dcp1A induces phosphorylation at position S315. Mutation of a number of ubiquitinated lysines to arginine in the C-terminal part of Dcp1A is associated with a defect in PB size, Dcp2 binding, and decapping activity. This suggests that both phosphorylation and ubiquitination are functionally linked. In addition, expression of a mutant ubiquitin results in complete loss of PBs (Tenekeci et al., 2016). These results indicate that ubiquitin plays a key role in PB formation, either independently or through modulation of other post-translational protein modifications like phosphorylation. However, the mechanisms by which post-translational protein modifications affect PB formation are not yet fully understood and deserve future analysis.

### 2.3 Neuronal RNA transport granules

Another type of cytoplasmic RNA granule, responsible for the intracellular transport of mRNA, are the neuronal RNA transport granules. Neurons have long neurites extending in multiple directions. RNA transcription takes place in the nucleus. Thus, mRNAs are transported to the place, where their encoded protein is needed, to be translated there (for example, at the synapse). Neurons regulate synaptic plasticity by spatiotemporal regulation of protein synthesis. In this way, the presence of a particular protein at a particular location in the right time is ensured. The neuronal RNA transport granules control this phenomenon by transporting mRNAs together with the translation machinery to its target site. During transport, the translation is inhibited, and at the final destination the translational block is released to allow the mRNA to be translated (Besse and Ephrussi, 2008; Gumy et al., 2011; Shigeoka et al., 2016). Neuronal RNA transport granules are transported along the microtubules to their final destination *via* the motor proteins, kinesins and dyneins (reviewed in (Urbanska et al., 2017; De Graeve and Besse, 2018)). Neuronal RNA granules are round shaped assemblies of tightly packed electron dense material of approximately 150–1,000 nm in size (reviewed in (Khandjian et al., 2009; Holt and Schuman, 2013)). Several studies confirmed the presence of translation initiation and elongation factors, and ribosomes in neuronal RNA transport granules. Further, these granules contain mRNAs that are blocked at the translation initiation step (Krichevsky and Kosik, 2001; Elvira et al., 2006; El Fatimy et al., 2016). One key protein that can function as translational repressor during RNA transport and regulate local protein synthesis and synaptic function is FMRP (reviewed in (Lai et al., 2020)). By time-lapse video microscopy, single granules were found to merge and form cargoes that are transported from the soma to distal locations. A substantial amount of FMRP target RNAs is transported in granules. Furthermore, FMRP is more abundant in granules than in polyribosomes. All these findings support a significant role for FMRP in granule-biology (El Fatimy et al., 2016).

Cells regulate the size, amount and composition of all types of RNA granules according to their metabolic priorities. RNA granules could be cell-type specific, like, neuronal RNA transport granules, or context-specific like the *de novo* paraspeckles that are generally absent but assemble during embryonic stem cell patterning (Chen and Carmichael, 2009). RNA granules are either present in all cell conditions or are induced by stress. Almost all RNA granules change their size, number, morphology, and composition in response to stress (reviewed in (Moujaber and Stochaj, 2018; An et al., 2019; An et al., 2021)).

### 2.4 Liquid-liquid phase separation

**FIGURE 3:**
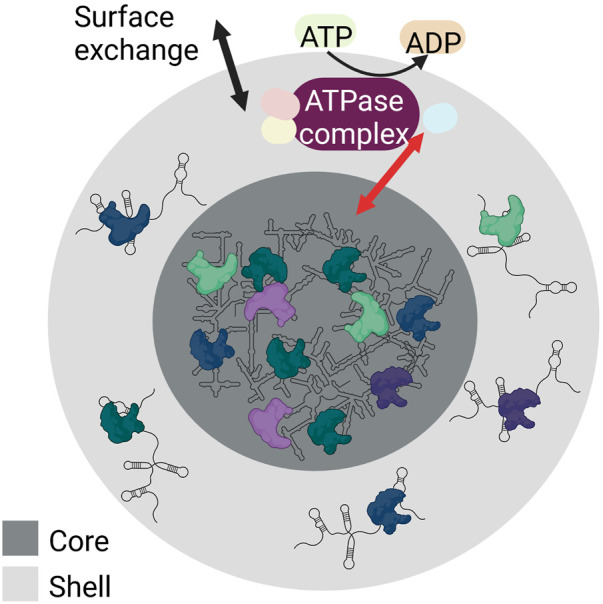
Schematic showing the internal structure and LLPS of RNA granules. RNA granules have a core (dark grey) made of condensed RNA-RNA and RNA-protein complexes, and a shell (light grey) containing RNA-RNA and RNA-protein complexes characterized by weak interactions. Both the shell and the core of the RNA granules build a dynamic system. Both surface exchange (black arrow) and core-shell transitions are possible (red arrow). These are modulated by numerous RNA-protein interactions and require ATP hydrolysis (yellow) as various ATPase complexes (purple) are involved that regulate various steps of granule assembly and stability. Created with BioRender.com.

The biogenesis of RNA granules occurs by liquid-liquid phase separation (LLPS). LLPS is the process of accumulating biomolecules into a dense phase by separating themselves from their surrounding light phase. Weak RNA-protein and RNA-RNA interactions aid LLPS. The droplets formed by LLPS are ball-shaped and can assemble or dissociate within seconds, thereby responding to signals quickly. This behaviour gives RNA granules the ability to assemble from smaller droplets that fuse, to condense, to dissolve, and to exchange content with their surroundings. Despite this flexibility, they are internally structured with a stable core structure and a more dynamic shell (reviewed in (Tian et al., 2020; An et al., 2021)) (Figure 3).

Granule formation and core-shell transitions are modulated by numerous RNA-protein interactions. These interactions require the hydrolysis of ATP to ADP by ATPases. In this process, different ATPase complexes are responsible for different steps of granule assembly, dynamics, and stability (Jain et al., 2016). For example, the ATPase-active dynein and its counterpart kinesin are required for the transport of ribonucleoproteins to granules (Loschi et al., 2009). The ATPase complexes MCM (mini-chromosome maintenance) and RVB (RuvB-like helicase) stabilize granules (Jain et al., 2016). In contrast, the ATP-dependent CCT (chaperonin-containing T) complex conserved in yeast and mammals can inhibit SG assembly.

For LLPS and the subsequent maintenance of RNA granules, both intrinsically disordered proteins (IDPs) and multivalent protein are required. While each type of RNA granule contains a certain signature of RNA-protein-content that distinguishes it from other granule types, some components like IDPs and multivalent proteins, are shared by different types of granules. Since there is a limited intracellular stock of these proteins critical for LLPS, the different types of granules compete for them. Thus, when there is an assemblage and expansion of one type of RNA granule, the other granules often shrink (Hochberg-Laufer et al., 2019; Rossi et al., 2020).

Important proteins that reside in RNA granules, e.g., IDPs, often have a susceptibility to misfold and undergo aberrant interactions. Therefore, proteins in RNA granules are often under close surveillance by the cellular protein quality control system. The protein quality control system involves chaperones that assist the conformational folding of proteins and cellular protein degradation pathways. Deregulation of the protein quality control system, for example in neurodegeneration, is thus often linked to aberrant RNA granules composition and dynamics. Those aberrant RNA granules can no longer execute their physiological functions and thus contribute to disease development (reviewed in (Zhang and Ashizawa, 2017; An et al., 2021)).

### 2.5 RNA granules in CXG repeat disorders

The appearance of aberrant RNA granules in CXG repeat expansion diseases was first reported more than 20 years ago, when intranuclear RNA granules, referred to as nuclear foci, where discovered in DM1. Here, the mutant CUG-expanded RNA encoding DMPK (Myotonic Dystrophy Protein Kinase) induces the assembly of nuclear foci in patient fibroblasts and muscle biopsies (Taneja et al., 1995). In line, also other CXG repeat containing RNAs induce formation of nuclear foci, e.g., the CAG-expanded RNAs HD or SCA3 (Ho et al., 2005; de Mezer et al., 2011). While historically, nuclear foci were the first aberrant RNA granules described in CXG repeat diseases, we will focus here on cytosolic RNA granules.

**FIGURE 4:**
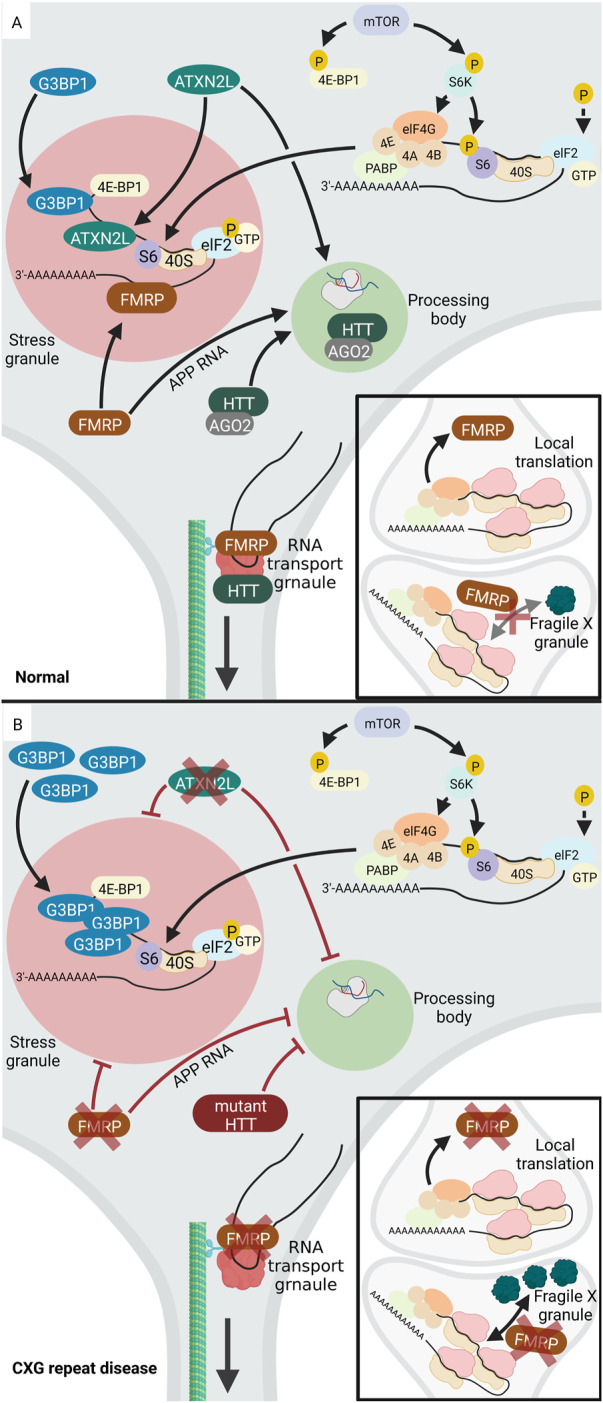
Altered RNA granule formation in CXG repeat diseases. **(A)** In normal cells, several proteins locate to and regulate RNA-processing in PBs (light green), including HTT, AGO2, ATXNL2. During a stress event, phosphorylation of elF2 (light blue) inhibits translation and the translationally silent RNA is organized into SGs (light red) *via* the activity of several RNA-binding proteins, e.g., FMRP, ATXNL2, or G3BP1. FMRP (brown) is involved in regulation of translation at the pre- and postsynapse. While FMRP blocks translation during transport, it needs to be released from the RNA to allow local synaptic translation. **(B)** Upon loss of function of FMRP (brown) in FXS, SG formation is inhibited. Similarly, loss of function of ATXNL2 (green) in SCA2 leads to disturbed SG formation. In HD, G3BP1 is overexpressed, leading to increased SG formation and PBs are inhibited. Under disease-conditions with a mutation or lack of HTT or ATXNL2, PB formation is reduced. While RNA-binding proteins like FMRP and HTT normally promote neuronal RNA transport granules, their loss of function decreases RNA transport. Upon loss of function of FMRP, the number of presynaptic Fragile X granules increases together with an increased local translation rate. Created with BioRender.com.

One example of a CXG repeat that causes the formation of aberrant cytoplasmic RNA granules is the mutant CGG repeat in the *FMR1* gene. This mutation is associated with FXS, which is characterized by intellectual and developmental disabilities. The *FMR1* gene encodes the RNA-binding FMRP protein, which regulates mRNA translation, localization, and stability (reviewed in (Richter and Zhao, 2021)). Normally, FMRP is found in all different cytosolic RNA granules, namely SGs, PBs, neuronal RNA transport granules and fragile X granules (Figure 4). Fragile X granules are axonal RNA granules and are composed of FMRP and four FMRP homologues, the Fragile X related (FXR) proteins (Akins et al., 2017a). During neuronal development, but also during adolescence in rodents, fragile X granules regulate the expression of the axonal proteome (Christie et al., 2009; Akins et al., 2017b). Interestingly, phosphorylation of FMRP leads to interaction with CAPRIN1, a protein that stimulates SG formation (Kim et al., 2019). This suggests a mechanism by which FMRP attaches to and pushes the formation of SGs upon post-translational modification. Thus, the phosphorylation status of FMRP is significant for its ability to regulate translation. Dephosphorylation of FMRP by protein phosphatase 2A (PP2A) triggers ubiquitination and degradation of FMRP (Nalavadi et al., 2012). This decreases FMRP levels and thereby releases its function in translational silencing of RNAs inside SGs.

In contrast, CCG repeat mutations in *FMR1* deregulate formation of cytoplasmic RNA granules. Expansion of the CGG repeat in the *FMR1* gene is associated with decreased expression and loss of function of FMRP and thus FXS. In the absence of FMRP or its mutation under disease conditions, SG formation process is inhibited. In addition, also the formation of fragile X granules is affected, with an increase in fragile X granules in the FRM1-knockout mice (Christie et al., 2009). Accordingly, it can be assumed that the number of fragile X granules may be reduced in FXS. FMRP also plays a role in PB formation. One known target mRNA of FMRP is the amyloid precursor protein (*APP*) RNA, which FMRP normally recruits into PBs thereby inhibiting its translation (Lee et al., 2010). APP regulates synaptic function and the proteolytic cleavage of APP releases neurotoxic Aβ, which is important in Alzheimer’s disease (reviewed in (Soria Lopez et al., 2019)). In FXS, mutant FMRP loses its function to organize *APP* RNA into PBs (Lee et al., 2010). It is tempting to speculate that overexpression of APP, which leads to neurodegeneration in Alzheimer’s disease (reviewed in (Hardy and Selkoe, 2002)), may occur in FXS. There are a few studies suggesting that APP and Aβ levels are deregulated in human FXS models, however, future work is needed to consolidate these findings (reviewed in (WCJ, 2019)).

Finally, missense mutations in FMRP result in reduced RNA transport granule formation in *Drosophila*, while simultaneously increasing their dynamics (Starke et al., 2022). Thus, impaired mRNA transport along the axon and dendrites represents another RNA granule-coupled role of FMRP that is affected in FXS.

In addition to the above-mentioned examples of aberrant RNA granules in FXS, other CXG repeat disorders also exhibit altered homeostasis of RNA granules. In HD, the mutant HTT protein interacts with SG-associated proteins and redistributes into SGs under endoplasmic reticulum (ER) stress. Furthermore, SG-associated proteins are overexpressed in cortices of HD mouse models and HD patients. These include G3BP1, which initiates SG assembly *via* multimerization. The granule density of G3BP1 is significantly increased in HD. Interestingly, in HD cortical neurons, the TDP43 (TAR DNA-binding protein) which is a nuclear RNA/DNA-binding protein, is mislocalized to SGs in cytoplasm of G3BP1 positive granules (SanchezNguyen et al., 2021).

Besides the deregulation of SGs, PBs are also affected in HD. Normally, the HTT protein co-localizes with Argonaute (AGO2) in PBs. AGO2 belongs to the AGO family and is a component of the small RNA-mediated gene silencing pathway (reviewed in), and HTT through association with AGO2 contributes to RNA-mediated gene silencing in PBs. Mouse striatal cells expressing mutant HTT exhibit fewer PBs, indicating that PB formation is affected in HD (Savas et al., 2008). AGO2 and HTT are also components of RNA transport granules and co-traffic with RNA along the microtubules in dendrites (Savas et al., 2010). In line, HTT co-localizes with microtubule motor proteins during dendritic transport of *β-actin* (*ACTB*) mRNA and reduction in the levels of HTT reduces the mRNA transport (Ma et al., 2011). This suggests that in HD, the mutant HTT protein may impact RNA transport granules and thus RNA transport to the neurites.

Like HD, SCA2 and SCA7 are also caused by expansion of a CAG repeat. The mutant CAG repeat is located in the *Ataxin-2* (*ATXN2*) gene in SCA2 and the *Ataxin-7* (*ATXN7*) gene in SCA7. As in HD, a correlation between disease and SG formation was discovered. The disease-causing protein in SCA2, ATXN2, has a paralog termed ataxin-2-like (ATXN2L), which is a component of SGs. Overexpression of ATXN2L leads to the induction of SGs, whereas low ATXN2L levels reduce the number and size of SGs. Therefore, ATXN2L can be considered as a SG regulator, probably *via* the interaction with G3BP1. Furthermore, overexpression or reduction of ATXNL2 impacts the presence of microscopically visible PBs. Thus, ATXNL2L is involved in the regulation of at least two types of RNA granules: SGs and PBs (Kaehler et al., 2012). In SCA7, the elongated ATXN7 protein affects the expression of G3BP1 and the behaviour of TDP43. In SCA7 cells, mutant ATXN7 aggregates both inside the nucleus and in the cytoplasm. TDP43 co-localizes with ATXN7 aggregates. In addition, SCA7 cells overexpresses G3BP1, which in turn leads to increased SG formation. Besides its localization in aggregates, ATXN7 also localizes to these SGs together with G3BP1 (Niss et al., 2022).

Additional examples of CAG repeat expansion diseases with altered RNA granule pattern are SCA6 and SCA8. SCA6 is caused by a CAG repeat expansion encoding polyglutamine in the *CACNA1A* gene (calcium voltage-gated channel subunit alpha1). Here, a reduced expression of BDNF (brain-derived neurotrophic factor) in SCA6 cerebellum and mislocalization of BNDF into aberrant dendritic granules, which are absent in controls, was observed (Takahashi et al., 2012). This study suggests that the aggregated polyglutamine protein interferes with normal BDNF trafficking in dendrites. Moreover, in SCA8, which is caused by a CAG (CUG on the opposite strand) repeat expansion mutation in the *ATXN8* gene (*ATXN8OS* on the opposite strand), aberrant cytoplasmic inclusions have been described. Within these aberrant cytosolic granules that were found in an SCA8 autopsy case, small granular particles, identical to ribosomes were found. However, future work is required to show in closer detail how the reverse or forward transcripts of the expanded CAG/CUG repeat affect aberrant aggregation of ribosomes and neurodegeneration (Yokoyama et al., 2014).

Another example of deregulated RNA granule in CXG repeat diseases is DM1, which is caused by expansion of a CUG repeat in *DMPK*. While the traditional cellular hallmark of the disease is the formation of nuclear foci containing expanded *DMPK* RNA, also formation of SGs and PBs is affected. Mutant CUG repeat *DMPK* RNA recruits RNA-binding proteins including muscleblind-like protein 1 (MBNL1) and CUG-binding protein 1 (CUGBP1). Both MBNL1 and CUGBP1 are SG and PB components. In cell culture models of DM1, the structure and regulation of SGs and PBs is altered (Gulyurtlu et al., 2022) (Figure 4). Moreover, while normally cell stress efficiently triggers formation of SGs, to which Staufen1 is recruited, DM1 myoblasts fail to properly form SGs upon oxidative stress. This blockage was not observed in disease-fibroblasts, which show lower expression and sequestration of toxic CUG repeat mRNAs then myoblasts, suggesting a cell type-specific defect. Down-regulation of Staufen1 in disease myoblasts normalizes SG formation, showing that Staufen1 is a key player in aberrantly reduced SG formation (Ravel-Chapuis et al., 2016). In contrast, in another study, it was seen that the expression of mutant CUG repeat RNA induced formation of SGs. These SGs trap the mRNA coding for MRG15, a DNA repair and DNA remodeling factor. This results in translational block and consequently lower MRG15 protein levels in CUG-expressing cells (Huichalaf et al., 2010). Together, these studies show that CUG-repeat expansion in DM1 can affect SG formation in two ways: pushing aberrant SG assembly and reducing the abnormal SG assembly-response to oxidative stress.

In summary, abnormal formation of intracellular RNA granules occurs in several CXG repeat expansion disorders. This includes the impaired formation of SGs, with both increased or decreased amounts of SGs, altered levels of PBs, alterations of RNA transport granules, and formation of aberrant RNA granule species. In addition to these irregular intracellular RNA vesicles, the quantity and appearance of EVs are also affected in CXG repeat expansion disorders.

## 3 Extracellular vesicles

**FIGURE 5:**
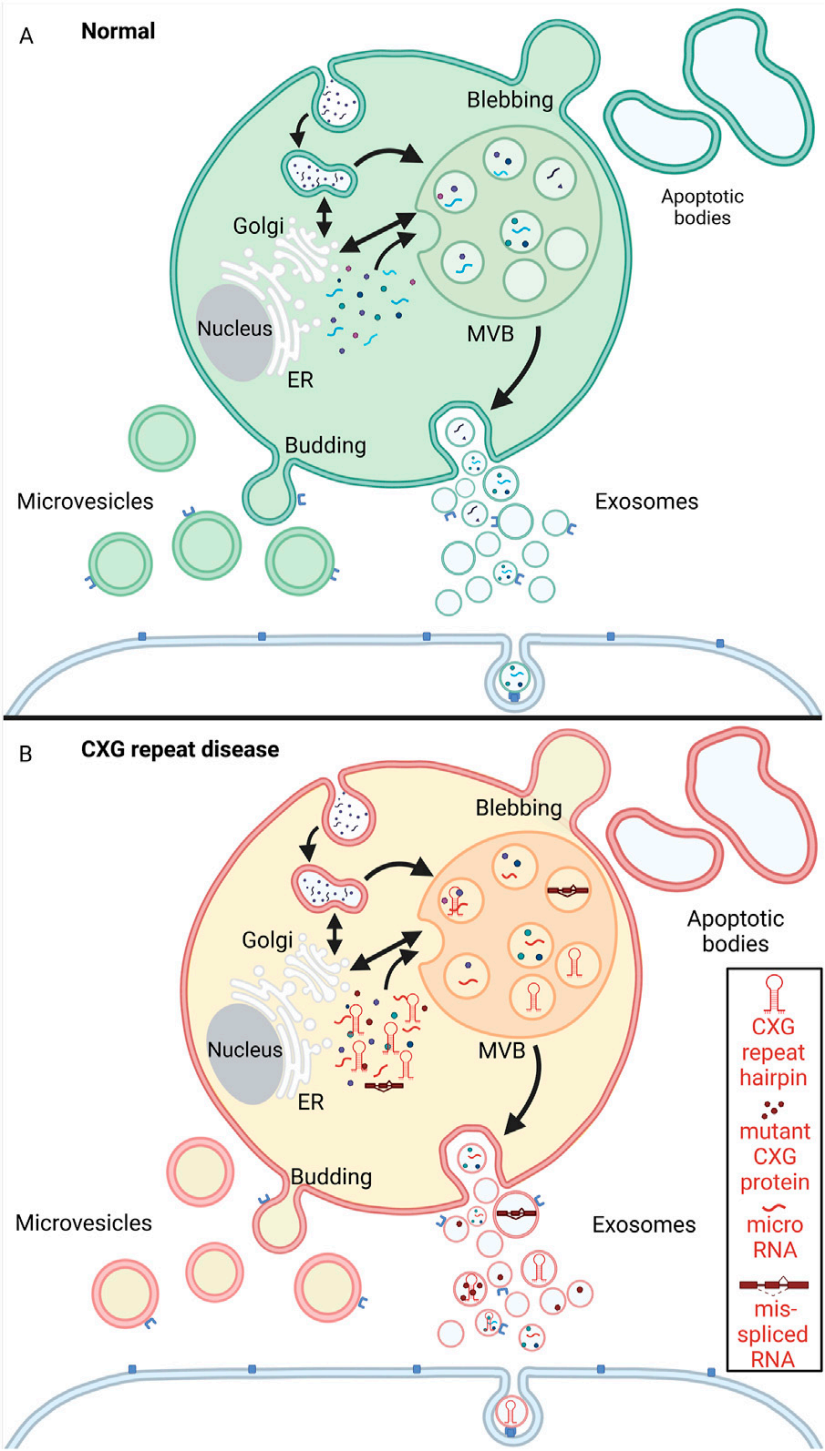
Schematic representation of extracellular vesicle biogenesis under physiological conditions (A, green) and CXG repeat disease cells (B, red/yellow). **(A)** Microvesicles are released to the extracellular space by budding from the plasma membrane. Apoptotic bodies arise from membrane protrusions (blebbing) during fragmentation of an apoptotic cell (ES, 2007; Akers et al., 2013; Povea-Cabello et al., 2017; Tricarico et al., 2017). Exosomes are formed by a multistep process that starts with endocytosis (the inward budding of the cell membrane and internalization of cargo into early endosomes). The early endosomes mature into multivesicular bodies (MVBs). By invagination of the MVB membrane intraluminal vesicles (ILVs) are formed. During this process, cytosolic components like proteins, RNAs and lipids are incorporated into ILVs. The ILVs are then released as exosomes from the MVBs into the extracellular space by fusion with the plasma membrane. The exosomes can then be taken up by neighboring cells *via* endocytosis. **(B)** In the context of CXG repeat diseases, pathogenic mutant RNA (hairpin, red), micro RNA, mis-spliced RNA and aggregating proteins (red dots) are passed on to neighboring cells *via* the exosomes. Created with BioRender.com.

Like RNA granules help connecting and regulating different intracellular mechanisms, cells release vesicles into the extracellular space. EVs participate in intercellular communication but also remove excess or unnecessary constituents from cells to maintain cellular homeostasis. Unlike the intracellular RNA granules, EVs are enclosed by a lipid bilayer. EV is an umbrella term that comprises all the vesicles in the extracellular space, namely exosomes, microvesicles (also called microparticles or ectosomes), and apoptotic bodies (also known as apoptosomes). While the apoptotic bodies could be regarded as the biggest of all EVs with a size ranging from 1–5 μM, microvesicles are ~100–1,000 nm large, making the 30–150 nm sized exosomes the smallest subpopulation (reviewed in (Théry et al., 2018; Battistelli and Falcieri, 2020; Nabariya et al., 2020)) (Figure 5).

Exosomes are spherical, cell-specific, nanosized EVs, which are secreted by almost all mammalian cells (reviewed in (Nabariya et al., 2020)). Within their lipid bilayer, exosomes encapsulate several biomolecules such as DNA, RNAs including mRNA and non-coding RNAs (transfer RNAs (tRNAs), ribosomal RNAs (rRNAs), microRNAs (miRNAs)) proteins (membrane, transmembrane, soluble proteins), as well as a wide range of peptides, lipids, and their derivatives. Though the presence of proteins in exosomes was reported just after the discovery of exosomes, the confirmation of the presence of RNA in exosomes and that the process of translation could take place inside the exosomes completely revolutionized the field of exosomal biology (Ratajczak et al., 2006; Valadi et al., 2007; Kalra et al., 2016). Interestingly, studies show the presence of several components of SGs in exosomes (Syed et al., 2020). Exosomes not only enable intercellular communication and biomolecule shuttling, but also the composition of the exosomes reflects the genetic, epigenetic and conditional composition of its mother cell (reviewed in (Kalra et al., 2016; Mashouri et al., 2019)).

Exosomal biogenesis is a multistep process. Their biogenesis involves endosome formation that starts with inward budding of the cell membrane and internalisation of cargo into early endosomes. The early endosomes mature into multivesicular bodies (MVBs). By invagination of the MVB membrane, intraluminal vesicles (ILVs) are formed within the MVBs. Through this process, ILVs recruit cytosolic components like proteins, RNAs, lipids, and other exosome cargoes. The ILVs are then released from the MVBs into the extracellular space by fusion with the plasma membrane. These released ILVs are termed exosomes (Théry et al., 2002; Poteryaev et al., 2010). The detailed pathways of exosomal biogenesis have been extensively reviewed elsewhere (Théry et al., 2002; Stuffers et al., 2009; HJH, 2010; Colombo et al., 2013; Kalra et al., 2016).

In summary, there are three different kinds of EVs: Exosomes, microvesicles and apoptotic bodies. While, apoptotic bodies are cleared by phagocytosis, exosomes and microvesicles play a vital role in intercellular communication and also help removing excess biomolecules from the cell. Besides supporting normal cell function and homeostasis, EVs are important in the development of neurodegenerative diseases.

### 3.1 Extracellular vesicles and CXG repeat disorders

While upon disease conditions, the aberrant formation of the cytoplasmic RNA granules could push neurons towards degeneration, exosomes can act both as a friend and a foe. On one hand they are involved in the progression of the disease, on the other hand their potential as a therapeutic system cannot be ignored. Exosomes are involved in the pathogenesis, propagation, and progression of several neurodegenerative diseases (reviewed in (Russo et al., 2012; Younas et al., 2022)). However, in the context of CXG repeat disorders, the number of studies is limited and more studies to understand the role of exosomes in the pathogenesis of CXG repeat disorders are the need of the hour. In HD, there is increasing evidence that EVs play a role in releasing disease-specific cargo and even transporting it between cells (Figure 5). Here, mutant gene products are the polyglutamine protein and the CAG repeat RNA. Both may be released from disease cells and may even be taken up by the surrounding cells. With respect to the occurrence of disease hallmarks like the formation of protein aggregates, cell-to-cell transmission of mutant gene products like aggregate-prone polyglutamine proteins is one important disease mechanism. Several studies support that polyglutamine protein can be released and taken up by cells:

First, cellular uptake of polyglutamine peptide (44Q) occurs both in *in-vitro* and *in-vivo.* Upon this internalisation of the polyglutamine peptide, cytoplasmic aggregation is induced in the receiving cells (Ren et al., 2009). In line, cell-to-cell transmission of mutant polyglutamine HTT protein is also well evidenced (Yang et al., 2002; Herrera et al., 2011; Costanzo et al., 2013; Pecho-Vrieseling et al., 2014). Further, studies confirm the propagation of mutant HTT to an unrelated tissue in three HD patients, who underwent foetal striatal tissue transplantation, where mutant HTT aggregation was observed in the transplanted tissue (Cicchetti et al., 2014). While all these studies provide evidence that cell-to-cell transmission of mutant polyglutamine HTT protein takes place, they do not explicitly identify the route of cell-to-cell transfer. However, EVs are likely involved, since additional studies suggest that mutant *HTT* gene products can propagate from one cell to another by taking up exosomes from the surroundings that contain polyglutamine HTT. For example, when the conditioned medium from HEK cells overexpressing mutant HTT is used for treating SH-SY5Y cells, the exogenous mutant HTT protein is detectable in treated cells within 5 days of medium exposure. Also, co-culturing of murine neuronal stem cells with exosomes derived from HD fibroblasts (containing 143 repeats) for 4 days resulted in the detection of mutant HTT aggregates in neurons. Additionally, upon intraventricular injection of exosomes from HD fibroblast in mice, mutant HTT was detectable in the striatum, and the mice developed an HD-like phenotype (Jeon et al., 2016).

**FIGURE 6:**
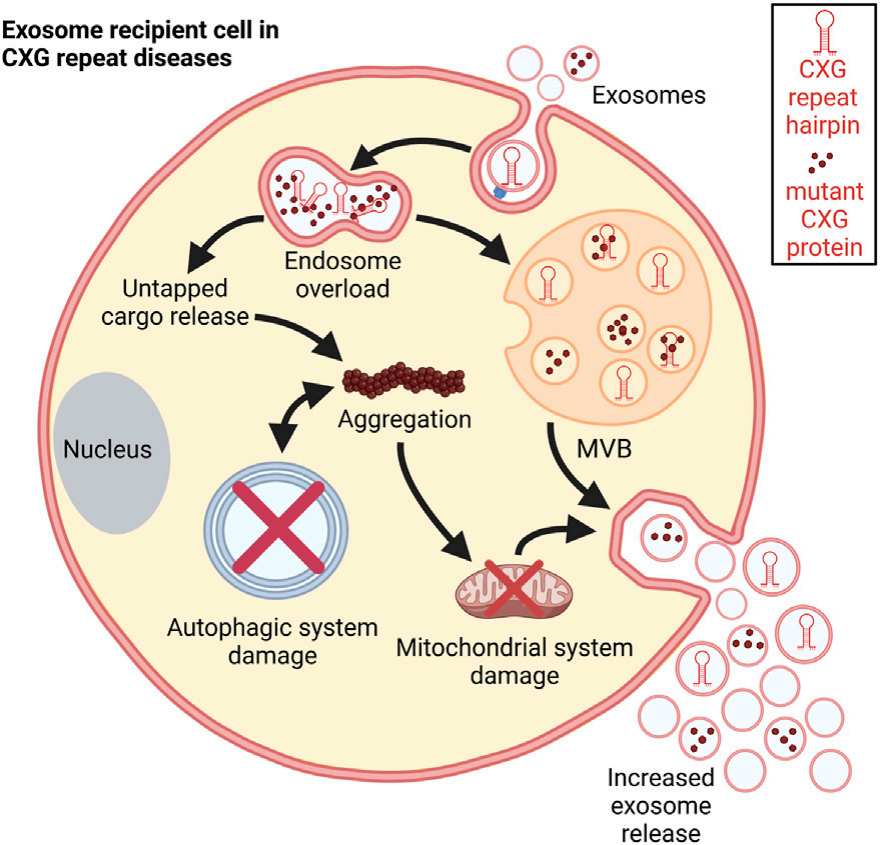
Schematic representation of the consequences of exosomal uptake. Post internalization, the disease-specific exosomal cargo overloads the endosome leading to untapped cargo release into the cytosol. This cargo could disrupt different cellular processes. It promotes aggregation and damages the mitochondrial system thereby increasing exosome release. Autophagic processes are impaired in CXG repeat disorders which facilitates further aggregation. These aggregations further push autophagic system collapse (Cortes and La Spada, 2014).

In HD cells, the EV secretion rate is substantially increased. This increase is linked to mitochondrial impairment. Mutant HTT enhances the activity of ca^2+^ channels and affects transcription. They together lead to functional deficits of mitochondria (reviewed in (Jin and Johnson, 2010)). HD cells secrete exosomes containing components of the mitochondrial machinery and elevated levels of mtDNA (Beatriz et al., 2022). Manifest HD patients show a sharp increase in EV release with autophagic and mitochondrial system damage (Figure 6). Autophagic flux (Pircs et al., 2018) impairment and accumulation of damaged mitochondria lead to an increased in the exosomal release and higher mitochondrial DNA levels in them (Beatriz et al., 2022).

Besides transferring mutant HTT protein, EVs also deliver the mutant CAG repeat RNA from one cell to another (Hong et al., 2017) (reviewed in (Ananbeh et al., 2021)). EVs that transferred mutant CAG repeat RNA induce HD symptoms and HD-like pathology in mice (Zhang et al., 2016).

While all these studies have been conducted on HD, EVs also play an important role in other CXG repeat expansion disorders. For example, there is evidence from studies on DM1 suggesting that a specific pattern of mis-splicing signatures can be detected in body fluids like blood or urine. These include 10 splicing variants in exosomal RNA that may serve as biomarker for the disease (Antoury et al., 2018). Furthermore, several exosomal muscle-specific miRNAs in serum of DM1 patients relating to muscle disease progress were identified (Koutsoulidou et al., 2017).

Moreover, there is evidence that another CXG repeat gene, *FMR1* that encodes FMRP, is connected to exosome loading. Specifically, FMRP controls exosomal cargo loading of miR-155 during inflammation (Wozniak et al., 2020). Thus, it is tempting to speculate that CXG repeat expansion, leading to decreased levels of functional FMRP, may affect miRNA loading into exosomes.

Taken together, CXG repeat expansion mutations can affect exosome content (Figure 6). The deregulation of vital RNA-binding proteins like FMRP and HTT leads to imbalanced loading of several components into exosomes setting a stage for the detection of several diagnostic biomarkers under disease-condition. On the other hand, the detection of the lower or higher expression levels of a particular biomolecules in the exosome might indicate a potential therapeutic aid.

### 3.2 Exosomes in diagnostics and therapy

Exosomes take part in several complex biological processes and play a vital role in the progression of several diseases. Since they are produced by virtually all cells, they are present in all biological fluids, and their molecular cargo is depictive of the physiological condition of the cell. For example, if a cell is under stress, the exosomes released could consist of several heat shock proteins and possibly some RNA granules. This molecular carriage of exosomes can be exploited as diagnostic tools to detect and monitor several diseases with minimum invasive diagnostic systems.

Additionally, exosomes are ideal candidates for the delivery of therapeutic molecules. Their lipid bilayer membrane protects their content, including enzymes, nucleic acids, and other biomolecules. The lipid bilayer further contains receptors that target exosomes to specific organs, tissues, or cells. Thus, exosomes represent a robust target-specific delivery system. Exosomes can be easily engineered due to their simple outer membrane composition and can easily cross the blood-brain barrier (reviewed in (Matsumoto et al., 2017)). EV secretion is conserved in many species and hence has a great potential as an inexpensive target specific delivery system (reviewed in (Kalra et al., 2016; Ahmed et al., 2022)).

In context to CXG repeat disorders, several studies have confirmed the compelling nature of exosomes both as diagnostic biomarkers and therapeutic systems. With regard to diagnostic biomarkers, HTT is detectable in EVs isolated from pig HD model as well as in HD patient plasma, with elevated HTT levels in EVs from plasma of HD groups when compared to controls suggesting that EV cargo may serve as biomarker for the disease (Ananbeh et al., 2022).

With respect to treatment options, several studies support that EVs may have therapeutic value. Of the many mechanisms that lead to neurodegeneration in HD, altered expression of the RE1-silencing transcription factor (REST) is one. While normally HTT represses REST, mutant HTT does not silence the activity of REST any longer. This results in transcriptional deregulation, e.g., the repression of REST target genes like *BDNF*. It is also known that in HD, the expression of *miR-124* is decreased, which leads to increased levels of the *miR-124* target gene REST. To compensate for the lack of miR-124 and thereby restore normal REST function, exosome-based delivery system called EXO-124 was developed. Therefore, exosomes were isolated from HEK293T cells overexpressing *miR-124*. Then EXO-124 was injected into the striatum of HD mice. While the REST expression level was successfully reduced, this treatment did not improve in the behavioural phenotype of the mice (Lee et al., 2017). Nevertheless, this study proves the efficiency of exosomes as a delivery system and makes room for several other studies with other miRNAs that have therapeutic potential.

In line with the concept of transferring small RNAs *via* exosomes, hydrophobically modified siRNAs (hsiRNA) targeting *HTT* mRNA can be loaded into exosomes *via* co-incubation without altering their shape, size, or integrity. These loaded vesicles can then be internalized by the primary cortical neurons and lead to dose-dependent *HTT* mRNA silencing (Didiot et al., 2016). This experiment proposes the efficacy of exosomes as a target-specific delivery system for small RNAs. With respect to HD, several studies suggested that small antisense oligonucleotides that lead to *HTT* silencing may be a valuable approach to reduce *HTT* expression. However, the clinical trials with antisense oligonucleotides were stopped, and future work is required to optimize these therapeutic approaches.^1^

In another study, exosomes were isolated from adipose-derived stem cells. Then cultures of neural stem cells from an HD mouse model were treated with these exosomes for 5 days. Strikingly, this treatment significantly reduced mutant HTT aggregation (Lee et al., 2016).

In a comparative dataset study of PubMed-articles containing co-citation of genes linked to either the protein interactome of normal HTT or the impact of mutant HTT-driven perturbation, two HD-relevant datasets were identified, one of which was exosome synaptic functions. This suggests that synaptic plasticity and exosomes are linked to each other, and their functions are altered in HD. Furthermore, the miRNA and protein cargo of exosomes was enriched in the datasets of HD and many known drug targets where found in the exosome databases indicating the therapeutic potential of exosomes (Wang et al., 2017).

In the central nervous system, astrocytes secrete diverse substances that support neuronal function and viability. With respect to neurodegenerative diseases, exosomes secreted from astrocytes carry heat shock proteins that can reduce the toxicity of misfolded proteins. Studies conducted on an HD mouse model showed that mutant HTT is not present in astrocytic exosomes but can decrease exosome secretion from astrocytes in HD mice. Mutant HTT decreases the exosome secretion by downregulating aB-crystallin levels. aB-crystallin is a small heat shock protein that is enriched in astrocytes and mediates exosome secretion, and its overexpression reverts the decreased exosome release in the striatum of HD mice. Interestingly, injection of exosomes derived from wildtype astrocytes into the striatum of HD mice lowers mutant HTT aggregates, suggesting strong therapeutic potential of astrocytic exosomes in HD (Hong et al., 2017).

Additional evidence that exosomes may carry positive factors to treat HD comes from a study in HD mice, that shared blood circulation with healthy mice through heterochronic parabiosis. Heterochronic parabiosis could ameliorate the HD pathology. Furthermore, in the same study, in an *in-vitro* HD model treated with exosomes isolated from blood, cell viability was improved and HTT aggregation was reduced. Overall, this study suggests that pathology of HD can be improved by treatment with exosomes from healthy blood (Lee et al., 2021).

While many studies on the impact of exosomes on CXG repeat expansion disorders focus on HD, also in SCA3, an autosomal dominant CAG repeat disorder caused by a CAG repeat expansion in the *ATXN3* gene, exosomes are involved. With respect to biomarkers, exosomal RNAs from the plasma and cerebrospinal fluid (CSF) of SCA3 patients and controls were isolated. They found one miRNA; *mir-7014*, that was upregulated in CSF exosomes and downregulated in plasma exosomes. Since exosomes can pass the blood–brain and blood–CSF barriers, these opposite changes in plasma- and CSF-derived exosomal *mir-7014* levels are to date not clear and deserves further analysis. Further, *mir-7014* level correlated with the age of onset, while no correlation with other clinical indexes, e.g., SARA scores, was found. Together these findings make *mir-7014* an interesting candidate as exosomal biomarker for SCA3 (Hou et al., 2019).

With respect to a putative therapeutic potential of exosomes in SCA3, a study of a SCA3 mouse model that was treated with exosomes isolated from healthy mesenchymal stem cells was conducted. Exosome treatment improved rotarod performance and attenuated the neuropathology, e.g., cerebellar myelin loss, Purkinje fibre loss and neuroinflammation, suggesting a great potential of exosome treatment as SCA3 therapy (Zhang et al., 2019; You et al., 2020).

In summary, exosomes are of great importance in CXG repeat expansions disorders: they carry disease-specific cargo, thereby enabling identification of biomarkers for disease progression, and they open the path to novel treatment options.

## 4 The RNA granule-exosome crosstalk

Exosomes and cytoplasmic RNA granules share similarities: both have a huge cargo of biomolecules including RNAs and proteins (reviewed in) and both originate from the cytoplasm. Interestingly, many RNA-binding proteins that are components of SGs or PBs (e.g., FMRP, IGF2BP1, YBX1, SYNCRIP, HNRNPA1, HNRNPA2B1, HNRNPU) are also associated with EV loading, suggesting a possible link between intracellular RNA granules and loading of EV-RNA (Markmiller et al., 2018; Leidal et al., 2020; Liu et al., 2021a) (reviewed in (Parolini et al., 2009; Mittelbrunn et al., 2011; Montecalvo et al., 2012; Haimovich et al., 2017; Wolozin and Ivanov, 2019)). For example, in HEK293 cells there is an 18.4% overlap of the PB proteome, and a 28.7% overlap of the SG proteome with the proteome of small EVs (Liu et al., 2021a). From the same study, there is evidence that LLPS of RNA-protein complexes into RNA granules concentrates these components for their subsequent sorting into EVs. In line with the concept that assembly of RNA-protein complexes *via* LLPS is important for sorting specific cargo into EVs, condensation of YBX1 into PBs by LLPS is necessary for sorting miRNAs into exosomes (Liu et al., 2021a).

It is tempting to speculate that neither single RNAs nor proteins are taken up from the cytoplasm into exosomes, but RNA-protein complexes segregate from RNA granules into exosomes. On average, cytoplasmic granules are larger than exosomes, meaning that RNA-protein complexes may separate from the large RNA granules, get internalised in the MVBs and finally get secreted as exosomes.

Once secreted from the cell, EVs can transfer their cargo including RNA transcripts to recipient cells. There are three potential ways of EV-RNA uptake: cell surface membrane fusion which is direct delivery of cargo into cytoplasm, cell contact-dependent mechanisms, e.g., *via* a synapse or *via* nanotube structures, or by endocytic pathways including micropinocytosis, phagocytosis, clathrin-mediated, caveolin-mediated, or receptor-mediated endocytosis (Mulcahy et al., 2014). The majority of uptake occurs *via* the endocytic pathways, by which the cargo is delivered inside endosomes and would face lysosomal degradation (Kanada et al., 2015; Hung and Leonard, 2016; Bonsergent et al., 2021). If the system is impaired, like in neurodegenerative disorders, the aberrant proteins and RNAs could escape the endolysosomal degradation system breaking the cell’s line of defence of the recipient cell. The recipient cells might sort these aberrant RNA transcripts and proteins into SGs or PBs, but this may lead to imbalanced intracellular RNA granule homeostasis (Dellar et al., 2022).

Taken together, there is clear evidence that exosome- and RNA granule-contents overlap. However, intensive studies are required to establish this link and understand the exact underlying molecular mechanisms of LLPS-linked conversion of these organelles into each other. Given the fact that RNA-protein interactions within both RNA-granule and exosomes are deregulated in CXG repeat expansion disorders, this gives a new vision to the way we look at the exosome-RNA granule’s intrinsic network and its role in disease development.

## 5 Conclusions and future perspectives

RNA function is tightly spatiotemporally regulated within the cell. Intracellular RNA granules are membrane-less organelles, in which RNA-protein complexes are condensed by LLPS. These granules have distinct function, ranging from transport of specific RNAs to their desired location, over silencing of translation as a reaction on cell stress, to RNA degradation. Some proteins like FMRP occur in different types of granules. In CXG repeat expansion diseases, the normal distribution of FMRP between the different types of granules is disturbed, leading to imbalance of RNA granules. Interestingly, post-translational modification of such regulator proteins like FMRP (e.g., phosphorylation or ubiquitination) plays a role in regulating these proteins’ function in granule formation. Thus, a key aspect of future studies should be to investigate the impact of phosphorylation or ubiquitination events on RNA-protein interactions as key steps for phase separation and granule assembly/disassembly.

Besides the LLPS-driven intracellular RNA-organization into granules to achieve spatiotemporal regulation of RNA-activity, RNA can also be transported intercellularly, and thus contribute to cell-cell communication. One route for this intercellular transport is *via* EVs. There is recent evidence that intracellular granules that are built *via* LLPS are precursors for EVs. Interestingly, like RNA granules assembly, also EV loading of miRNA cargo is regulated by ubiquitination and phosphorylation events: Ubiquitination of RNA-binding proteins, e.g., YBX1 helps selecting miRNAs for EV loading. Stress induced phosphorylation of Caveolin-1 leads to altered EV-miRNA-content (Palicharla and Maddika, 2015; Shurtleff et al., 2017; Lee et al., 2019).

Future work to directly compare the RNA content of intracellular RNA granules and EVs may promote a better understanding of RNA packaging and vesicle-release mechanisms. Furthermore, it will show if the number and identity of intracellular granules affects the composition of exosomes.

In this context consideration of the heterogeneity of EVs is of critical importance. Recently, it was found that a light subpopulation of small EVs contained around nine-fold less RNA per EV than a dense subpopulation (Barman et al., 2020). Thus, EV sub-types may derive from different intracellular locations that contain different RNA-protein complexes/RNA granule types. Future studies of the RNA loading processes into EVs are needed to substantiate this.

Disease specific cell content including protein aggregates as well as mutant transcripts can transmit to unaffected cells *via* EVs, leading to spreading of disease phenotypes (e.g., protein misfolding). The uptake of cellular cargo *via* EVs depends on receptor-ligand interactions. Interestingly, recent studies show that the presence of viral proteins (e.g., vesicular stomatitis virus glycoprotein and SARS-CoV-2 spike S) increases uptake of EV-cargo that is important for neurodegenerative diseases (e.g., prion-diseases of tauopathies) (Liu et al., 2021b). These data raise the possibility that viral infections may facilitate intercellular transfer of disease-specific cargo *via* EVs. Thus, future studies should address the importance of viral infections for disease progression of CXG diseases.

The above-mentioned mechanism of transmission of pathogenic particles *via* exosomes as well as RNA toxicity in general also occur in C9orf72 repeat expansion mutations (reviewed in (McEachin et al., 2020)) (Westergard et al., 2016; Frottin et al., 2021). Unlike the triplet expansion in CXG diseases, the disease pattern is a hexanucleotide repeat expansion associated with Amyotrophic lateral sclerosis (ALS). Interestingly, an RNA exosome complex can also lead to the degradation of expanded hexanucleotide repeat RNA in C9orf72 (Kawabe et al., 2020e1027). A similarity between these disease groups is the impairment of RNA processing events. For instance, through binding of RNA binding Proteins (reviewed in (Kumar et al., 2017)). Coming to stress granules, in ALS, the production of dipeptide repeats, specifically poly-(Gly-Arg) and poly-(Pro-Arg) induce nucleolar stress and impaired stress granule formation (Tao et al., 2015). Changes related to SG have been known before (Li et al., 2000) (reviewed in (Dewey et al., 2012)). Taken together, this implies an important role of aberrant stress granule assembly during cellular stress in ALS (Kwon et al., 2014; Wen et al., 2014; Tao et al., 2015) (reviewed in (Buchan, 2014)). Further, C9ORF72 can be found inside of PBs and be recruited to SGs upon stress-related stimuli (Thomas et al., 2009; Maharjan et al., 2017). In summary, the disease patterns of CXG repeat diseases and C9ORF72 repeat diseases are similar in terms of symptoms, type of mutation (repeat expansion), as well as deregulated cellular mechanisms. TDP43 is also mislocalized in the neurodegenerative disease amyotrophic lateral sclerosis (ALS) and leads to aggregation (Peters and Meister, 2007; Chew et al., 2019).

EVs have a great potential as both diagnostic biomarkers and therapeutic application system to deliver specific cargo. Several studies have found that treatment of disease models with normal exosomes significantly reduces disease symptoms both *in-vitro* and *in-vivo* (Lee et al., 2016). Moreover, exosomes could be effortlessly engineered to carry drugs or other biomolecules like miRNAs for RNA silencing. Additionally, differences in exosomal RNA cargo (e.g., miRNAs or specific splice variants) in CXG repeat expansion diseases may present robust diagnostic markers. However, there are many challenges around this field of research, which need to be tackled before this system actually comes into diagnostic and therapeutic use.

In conclusion, in CXG repeat disorders, the mutant CXG repeat RNA folds into hairpin structures that affect normal RNA-protein interaction networks. This leads to an abnormal formation of intracellular RNA granules in CXG repeat disorders. Here, the function of SGs, PBs, and neuronal RNA transport granules is hindered. Exosomes, on the other hand are secreted from cells to the extracellular space. Its cargo is largely tampered in CXG repeat disorders. Interestingly, the cargo of RNA granules and exosomes overlap indicating the contact of RNA granules to the packaging system of exosomes. Extensive studies are requisite for the greater understanding of the RNA granule -exosome interplay and its significance for disease development.

## 6 Methods of isolation of RNA organelles and exosomes and markers for their characterization

An important aspect in research on RNA granules and EVs is their isolation and characterization. In this section we will summarize frequently used isolation techniques and markers for RNA granules and EVs. To date, several studies accentuate the role of cytoplasmic RNA granules in several diseases. However, the isolation and characterization procedures are diverse. Table 2 and Table 3 provide a summary of the literature of various isolation protocols and markers for characterization of different cytoplasmic RNA granules. It is of prime importance to understand that some proteins are shared between different granules. For example, Staufen1 which is a neuronal RNA transport granule marker, is also found in SGs. It is recruited there to help in recovering from the stress and dissolving the SG. Also, upon overexpression, proteins like Staufen1, Staufen2, FMRP, SMN, CPEB and pumilio2 could be detected in SGs (reviewed in (Anderson and Kedersha, 2006)).

**TABLE 2:** List of literature of various types of isolation procedures of SGs, PBs, neuronal RNA Transport granules, and RNA granules in general.

Type of granule	Type of technique	References
Stress granules	Differential ultracentrifugation	Jain et al. (2016)
	Differential ultracentrifugation + affinity purification	Khong et al. (2017)
		Wheeler et al. (2017)
		Hubstenberger et al. (2017)
Processing bodies	Differential ultracentrifugation + fluorescence activated particle sorting (FAPS)	Jønson et al. (2007)
Neuronal RNA transport granules	Differential ultracentrifugation + affinity purification	(Maher-Laporte et al., 2010) (Rat brain)
	Sucrose gradient ultracentrifugation (Isolation from brain homogenate)	Brendel et al. (2004)
	Sucrose gradient ultracentrifugation + immunoprecipitation (Isolation from brain homogenate)	Fritzsche et al. (2013)
	Differential ultracentrifugation + density gradient ultracentrifugation + affinity purification	Namkoong et al. (2018)
Mixed pellet	differential ultracentrifugation	Elvira et al. (2006)

**TABLE 3:** List of different markers for different cytoplasmic granules.

Type of granule	Markers	References
Stress granules	p-eIF2a	(reviewed in (Anderson and Kedersha, 2006; Buchan and Parker, 2009; Protter and Parker, 2016; An et al., 2021; Kedersha et al., 2013; Kedersha et al., 2005))
	eIF3, p116	
	eIF4AI	
	G3BP-1	
	PABP-1	
	TIA1	
	Ataxin-2	
Processing bodies	DCP1a	(reviewed in (Kedersha and Anderson, 2007; Luo et al., 2018; An et al., 2021))
	GE-1/hedls	
	4E-T	
	P54/RCK	
	Xrn1	
Stress dependent SGs and PBs	eIF4E	(reviewed in (Kedersha and Anderson, 2007))
	Lsm1	
	YB-1	
Neuronal RNA transport granules	FMRP	(reviewed in (Kiebler and Bassell, 2006; Pushpalatha and Besse, 2019))
	Staufen	
	SYNCRIP	
	Molecular motors	

Exosomes have been isolated from various biological sources including blood, saliva, cerebrospinal fluid, amniotic fluid, milk, tissues, cells, and cell culture medium (reviewed in (Sharma and Gimzewski, 2012; Kalra et al., 2016)) (Yenuganti et al., 2022). The increasing understanding of exosomes led to a growing research interest and different groups established their individual experiments for isolating and validating exosomes. This resulted in a lack of reproducibility and comparability. Therefore, in 2012, the International Society for Extracellular Vesicles (ISEV) was initiated to standardize the system. In 2014, they published a set of guidelines to standardize parameters that must be taken into consideration when portraying strong conclusions about specific subpopulations of EVs (exosomes in particular) (reviewed in (Lötvall et al., 2014; Théry et al., 2018)).

An overview of different methods of isolation of EVs is given in Table 4. Most commonly, differential ultracentrifugation is used for the isolation of EVs and/or exosomes in combination with different other techniques to more efficiently obtain specific EVs. These techniques include ultrafiltration, chromatographic techniques, or density gradient ultra-centrifugations. According to the MISEV (Minimal Information for Studies of Extracellular Vesicles), a minimum three positive markers including one transmembrane/lipid bound protein and one cytosolic protein and in addition a negative marker need to be stained to clearly identify exosomes in fractions of EVs. The list of different positive and negative markers has been summarized in Table 5. The most common markers to identify exosomes among the EV subtypes include Alix, TSG101, CD63, CD81 and CD9 (Keerthikumar et al., 2015; Minciacchi et al., 2015) (reviewed in (Mathivanan et al., 2010)). In addition to staining positive and negative markers, the isolated samples need to be analysed with two other techniques like electron microscopy, scanning-probe microscopy (SPM) including atomic-Force microscopy (AFM), or super-resolution microscopy, fluorescence correlation spectroscopy (FCS), high-resolution flow cytometry, or single particle analysis (reviewed in (Théry et al., 2018)).

**TABLE 4:** This table summarizes various methods to isolate exosomes.

Recovery vs specificity grid	Method of isolation	References/links for products/short description
High recovery, low specificity	Precipitation kits/polymers like PEG	(Karttunen et al., 2018)^a^
	low molecular weight cut off centrifugal filters with no further separation step	^b^ ^,^ ^c^
	Lengthy and/or high-speed ultracentrifugation without the low-speed centrifugal steps	In this protocol, we ultracentrifuge at 100000 X g for 70 min directly without the centrifugation steps to remove high molecular weight components and apoptotic bodies
intermediate recovery, intermediate specificity	Size Exclusion Chromatography	(Böing et al., 2014) (reviewed in (Liangsupree et al., 2021))
	high molecular weight centrifugal filters	Vergauwen et al. (2017)
	differential ultracentrifugation using intermediate time/speed with or without wash	Enderle et al. (2015)
	Density gradient ultracentrifugation	Li et al. (2018)
	Membrane Affinity Columns	^d^
low recovery, high specificity	Filtration combined with SEC to eliminate non-EV components	—
	Immuno–or other affinity isolations including Flow cytometry for large particles	(Maia et al., 2020)^e^

^a^ Capturem™ Extracellular Vesicle Isolation Kit (Mini) (https://www.takarabio.com/products/gene-function/cell-biology-assays/extracellular-vesicle-isolation) (Last accessed on 21/07/2022).

^b^ Steriflip^®^ filter units (https:// www.merckmillipore.com/DE/de/product/Steriflip-Filter-Units,MM_NF-C3238?CatalogCategoryID = ) (Last accessed on 21/07/2022).

^c^ Amicon^®^ Ultra Centrifugal Filters (https://www.merckmillipore.com/DE/de/life-science-research/protein-sample-preparation/protein-concentration/amicon-ultra-centrifugal/filters/YKSb.qB.GXgAAAFBTrllvyxi,nav) (Last accessed on 21/07/2022).

^d^ Strep-tag^®^ technology for exosome isolation (https://www.iba-lifesciences.com/strep-tag/exosome-isolation/) (Last accessed on 21/07/2022).

^e^ Izon Science/qEV Isolation and RNA/qEVsingle (https://www.izon.com/qev/overview?utm_term=izon%20qev&utm_campaign=qEV+Columns&utm_source=adwords&utm_medium=ppc&hsa_acc=1797985297&hsa_cam=17384675247&hsa_grp=136914957266&hsa_ad=601530194304&hsa_src=g&hsa_tgt=kwd1664089551290&hsa_kw=izon%20qev&hsa_mt=p&hsa_net=adwords&hsa_ver=3&gclid=Cj0KCQjwz96WBhC8ARIsAATR25393elYVNV-rsz33VsBiCXpa9g__quKYJTN5Pyo0MTnln7uI-M-yTQaAomNEALw_wcB) (Last accessed on 21/07/2022).

**TABLE 5:** This table summarizes different markers by which EVs could be characterized (reviewed in (Théry et al., 2018)). According to MISEV, at least three positive markers, including a transmembrane/lipid bound protein and cytosolic protein, and one negative marker should be shown to characterize exosomes in the EV fraction along with data from two other techniques like electron/atomic force microscopy and single particle analysis etc (Théry et al., 2018).

Type of protein	Specificity	Markers
Transmembrane/GPI anchored proteins (exosomal marker)	Non-tissue specific (ubiquitously expressed)	Tetraspanins: CD63, CD81, CD82
		Multi-pass membrane proteins: CD47, MHC class I, Integrins, transferrin receptor (TFR2)
		Complement binding proteins CD55 and CD59
	Cell/tissue specific	Epithelial cell: TSPAN8
		Leukocytes: CD37 and CD53
		Endothelial cells: PECAM1
		Breast Cancer: ERBB2
		T-Cells: CD3
		Neurons: Acetylcholinesterase/AChE-S, Amyloid beta A4/APP
Cytosolic proteins (exosomal marker)>	Protein binding ability (lipid or membrane)	ESCRT- I/II/III (TSG101)
		Accessory proteins: Alix, Flotillins 1 and 2, annexins, Heat shock proteins Hsc70 and Hsp84, Tau
—	Untapped integration in EVs	Hsp70, Actin, Tubulin, GAPDH
Negative markers	Plasma/serum EVs/EVs from cells cultured in bovine serum	Apolipoproteins A1/2 and B, albumin.

## Footnotes

1 Deeprose, C. (July 2021) Looking Back, Looking Forward. (http://www.ehdn.org/wp-content/uploads/2021/07/EHDN-NL-Jul2021-final.pdf) (last accessed on 19/07/2022).

## References

[B1] Ahmed F. Tamma M. Pathigadapa U. Reddanna P. Yenuganti V. R. (2022). Drug loading and functional efficacy of cow, buffalo, and goat milk-derived exosomes: A comparative study. Mol. Pharm. 19 (3), 763–774. 10.1021/acs.molpharmaceut.1c00182 35195427

[B2] Akers J. C. Gonda D. Kim R. Carter B. S. Chen C. C. (2013). Biogenesis of extracellular vesicles (EV): Exosomes, microvesicles, retrovirus-like vesicles, and apoptotic bodies. J. Neurooncol. 113 (1), 1–11. 10.1007/s11060-013-1084-8 23456661 PMC5533094

[B3] Akins M. R. Berk-Rauch H. E. Kwan K. Y. Mitchell M. E. Shepard K. A. Korsak L. I. (2017). Axonal ribosomes and mRNAs associate with fragile X granules in adult rodent and human brains. Hum. Mol. Genet. 26 (1), 192–209. 10.1093/hmg/ddw381 28082376 PMC5815656

[B5] Al-Mahdawi S. Pinto R. M. Ismail O. Varshney D. Lymperi S. Sandi C. (2008). The Friedreich ataxia GAA repeat expansion mutation induces comparable epigenetic changes in human and transgenic mouse brain and heart tissues. Hum. Mol. Genet. 17 (5), 735–746. 10.1093/hmg/ddm346 18045775

[B6] Almaguer-Mederos L. E. Falcón N. S. Almira Y. R. Zaldivar Y. G. Almarales D. C. Góngora E. M. (2010). Estimation of the age at onset in spinocerebellar ataxia type 2 Cuban patients by survival analysis. Clin. Genet. 78 (2), 169–174. 10.1111/j.1399-0004.2009.01358.x 20095980

[B7] An H. de Meritens C. R. Shelkovnikova T. A. (2021). Connecting the “dots”: RNP granule network in health and disease. Biochim. Biophys. Acta. Mol. Cell Res. 1868 (8), 119058. 10.1016/j.bbamcr.2021.119058 33989700

[B8] An H. Tan J. T. Shelkovnikova T. A. (2019). Stress granules regulate stress-induced paraspeckle assembly. J. Cell Biol. 218 (12), 4127–4140. 10.1083/jcb.201904098 31636118 PMC6891081

[B9] Ananbeh H. Novak J. Juhas S. Juhasova J. Klempir J. Doleckova K. (2022). Huntingtin Co-isolates with small extracellular vesicles from blood plasma of TgHD and KI-HD pig models of huntington's disease and human blood plasma. Int. J. Mol. Sci. 23 (10), 5598. 10.3390/ijms23105598 35628406 PMC9147436

[B10] Ananbeh H. Vodicka P. Kupcova Skalnikova H. (2021). Emerging roles of exosomes in huntington's disease. Int. J. Mol. Sci. 22 (8), 4085. 10.3390/ijms22084085 33920936 PMC8071291

[B11] Anderson P. Kedersha N. (2006). RNA granules. J. Cell Biol. 172 (6), 803–808. 10.1083/jcb.200512082 16520386 PMC2063724

[B12] Anderson P. Kedersha N. (2009). RNA granules: Post-transcriptional and epigenetic modulators of gene expression. Nat. Rev. Mol. Cell Biol. 10 (6), 430–436. 10.1038/nrm2694 19461665

[B13] Antoury L. Hu N. Balaj L. Das S. Georghiou S. Darras B. (2018). Analysis of extracellular mRNA in human urine reveals splice variant biomarkers of muscular dystrophies. Nat. Commun. 9 (1), 3906. 10.1038/s41467-018-06206-0 30254196 PMC6156576

[B14] Arimoto K. Fukuda H. Imajoh-Ohmi S. Saito H. Takekawa M. (2008). Formation of stress granules inhibits apoptosis by suppressing stress-responsive MAPK pathways. Nat. Cell Biol. 10 (11), 1324–1332. 10.1038/ncb1791 18836437

[B15] Arrasate M. Finkbeiner S. (2012). Protein aggregates in Huntington's disease. Exp. Neurol. 238 (1), 1–11. 10.1016/j.expneurol.2011.12.013 22200539 PMC3909772

[B16] Barman B. Ping J. Krystofiak E. Allen R. Prasad N. Vickers K. (2020). Biogenesis of RNA-containing extracellular vesicles at endoplasmic reticulum membrane contact sites. 10.1016/j.devcel.2022.03.012 PMC9075344 35421371

[B17] Battistelli M. Falcieri E. (2020). Apoptotic bodies: Particular extracellular vesicles involved in intercellular communication. Biology 9 (1), 21. 10.3390/biology9010021 31968627 PMC7168913

[B18] Beatriz M. Vilaça R. Anjo S. I. Manadas B. Januário C. Rego C. A. (2022). Defective mitochondrial-lysosomal axis promotes extracellular vesicles release of mitochondrial components in Huntington’s Disease. 10.1002/jex2.65 PMC11080813 38939215

[B19] Besse F. Ephrussi A. (2008). Translational control of localized mRNAs: Restricting protein synthesis in space and time. Nat. Rev. Mol. Cell Biol. 9 (12), 971–980. 10.1038/nrm2548 19023284

[B20] Biamonti G. Vourc'h C. (2010). Nuclear stress bodies. Cold Spring Harb. Perspect. Biol. 2 (6), a000695. 10.1101/cshperspect.a000695 20516127 PMC2869524

[B21] Böing A. N. van der Pol E. Grootemaat A. E. Coumans F. A. Sturk A. Nieuwland R. (2014). Single-step isolation of extracellular vesicles by size-exclusion chromatography. J. Extracell. Vesicles 3, 23430. 10.3402/jev.v3.23430 PMC4159761 25279113

[B22] Bonsergent E. Grisard E. Buchrieser J. Schwartz O. Théry C. Lavieu G. (2021). Quantitative characterization of extracellular vesicle uptake and content delivery within mammalian cells. Nat. Commun. 12 (1), 1864. 10.1038/s41467-021-22126-y 33767144 PMC7994380

[B23] Brendel C. Rehbein M. Kreienkamp H. J. Buck F. Richter D. Kindler S. (2004). Characterization of Staufen 1 ribonucleoprotein complexes. Biochem. J. 384, 239–246. 10.1042/BJ20040812 15303970 PMC1134106

[B24] Buchan J. R. (2014). mRNP granules. Assembly, function, and connections with disease. RNA Biol. 11 (8), 1019–1030. 10.4161/15476286.2014.972208 25531407 PMC4615263

[B25] Buchan J. R. Parker R. (2009). Eukaryotic stress granules: The ins and outs of translation. Mol. Cell 36 (6), 932–941. 10.1016/j.molcel.2009.11.020 20064460 PMC2813218

[B26] Chen L. L. Carmichael G. G. (2009). Altered nuclear retention of mRNAs containing inverted repeats in human embryonic stem cells: Functional role of a nuclear noncoding RNA. Mol. Cell 35 (4), 467–478. 10.1016/j.molcel.2009.06.027 19716791 PMC2749223

[B27] Chew J. Cook C. Gendron T. F. Jansen-West K. Del Rosso G. Daughrity L. M. (2019). Aberrant deposition of stress granule-resident proteins linked to C9orf72-associated TDP-43 proteinopathy. Mol. Neurodegener. 14 (1), 9. 10.1186/s13024-019-0310-z 30767771 PMC6377782

[B28] Christie S. B. Akins M. R. Schwob J. E. Fallon J. R. (2009). The FXG: A presynaptic fragile X granule expressed in a subset of developing brain circuits. J. Neurosci. 29 (5), 1514–1524. 10.1523/JNEUROSCI.3937-08.2009 19193898 PMC2746438

[B29] Chung M. Y. Ranum L. P. Duvick L. A. Servadio A. Zoghbi H. Y. Orr H. T. (1993). Evidence for a mechanism predisposing to intergenerational CAG repeat instability in spinocerebellar ataxia type I. Nat. Genet. 5 (3), 254–258. 10.1038/ng1193-254 8275090

[B30] Cicchetti F. Lacroix S. Cisbani G. Vallières N. Saint-Pierre M. St-Amour I. (2014). Mutant huntingtin is present in neuronal grafts in Huntington disease patients. Ann. Neurol. 76 (1), 31–42. 10.1002/ana.24174 24798518

[B31] Coller J. Parker R. (2005). General translational repression by activators of mRNA decapping. Cell 122 (6), 875–886. 10.1016/j.cell.2005.07.012 16179257 PMC1853273

[B32] Colombo M. Moita C. van Niel G. Kowal J. Vigneron J. Benaroch P. (2013). Analysis of ESCRT functions in exosome biogenesis, composition and secretion highlights the heterogeneity of extracellular vesicles. J. Cell Sci. 126, 5553–5565. 10.1242/jcs.128868 24105262

[B33] Cortes C. J. La Spada A. R. (2014). The many faces of autophagy dysfunction in huntington's disease: From mechanism to therapy. Drug Discov. Today 19 (7), 963–971. 10.1016/j.drudis.2014.02.014 24632005 PMC4096219

[B34] Costanzo M. Abounit S. Marzo L. Danckaert A. Chamoun Z. Roux P. (2013). Transfer of polyglutamine aggregates in neuronal cells occurs in tunneling nanotubes. J. Cell Sci. 126, 3678–3685. 10.1242/jcs.126086 23781027

[B35] De Graeve F. Besse F. (2018). Neuronal RNP granules: From physiological to pathological assemblies. Biol. Chem. 399 (7), 623–635. 10.1515/hsz-2018-0141 29641413

[B36] de Mezer M. Wojciechowska M. Napierala M. Sobczak K. Krzyzosiak W. J. (2011). Mutant CAG repeats of Huntingtin transcript fold into hairpins, form nuclear foci and are targets for RNA interference. Nucleic Acids Res. 39 (9), 3852–3863. 10.1093/nar/gkq1323 21247881 PMC3089464

[B37] Decker C. J. Burke J. M. Mulvaney P. K. Parker R. (2022). RNA is required for the integrity of multiple nuclear and cytoplasmic membrane-less RNP granules. EMBO J. 41 (9), e110137. 10.15252/embj.2021110137 35355287 PMC9058542

[B38] Decker C. J. Parker R. (2012). P-Bodies and stress granules: Possible roles in the control of translation and mRNA degradation. Cold Spring Harb. Perspect. Biol. 4 (9), a012286. 10.1101/cshperspect.a012286 22763747 PMC3428773

[B39] Dellar E. B. L, L. A. Hill C. Melling G. E. Carter D. R. F. Baena‐Lopez L. A. (2022). Unpacking extracellular vesicles: RNA cargo loading and function. J. Extracell. Bio. 1 (5), e40. 10.1002/jex2.40 PMC11080855 38939528

[B40] Dewey C. M. Cenik B. Sephton C. F. Johnson B. A. Herz J. Yu G. (2012). TDP-43 aggregation in neurodegeneration: Are stress granules the key? Brain Res. 1462, 16–25. 10.1016/j.brainres.2012.02.032 22405725 PMC3372581

[B41] Didiot M. C. Hall L. M. Coles A. H. Haraszti R. A. Godinho B. M. Chase K. (2016). Exosome-mediated delivery of hydrophobically modified siRNA for huntingtin mRNA silencing. Mol. Ther. 24 (10), 1836–1847. 10.1038/mt.2016.126 27506293 PMC5112038

[B42] DS (2017). Cajal bodies and snRNPs - friends with benefits. RNA Biol. 14 (6), 671–679. 27627834 10.1080/15476286.2016.1231359 PMC5519240

[B44] El Fatimy R. Davidovic L. Tremblay S. Jaglin X. Dury A. Robert C. (2016). Tracking the fragile X mental retardation protein in a highly ordered neuronal RiboNucleoParticles population: A link between stalled polyribosomes and RNA granules. PLoS Genet. 12 (7), e1006192. 10.1371/journal.pgen.1006192 27462983 PMC4963131

[B45] Elvira G. Wasiak S. Blandford V. Tong X. K. Serrano A. Fan X. (2006). Characterization of an RNA granule from developing brain. Mol. Cell. Proteomics 5 (4), 635–651. 10.1074/mcp.M500255-MCP200 16352523

[B46] Enderle D. Spiel A. Coticchia C. M. Berghoff E. Mueller R. Schlumpberger M. (2015). Characterization of RNA from exosomes and other extracellular vesicles isolated by a novel spin column-based method. PloS one 10 (8), e0136133. 10.1371/journal.pone.0136133 26317354 PMC4552735

[B47] ES (2007). Apoptosis: A review of programmed cell death. Toxicol. Pathol. 35 (4), 495–516. 17562483 10.1080/01926230701320337 PMC2117903

[B48] Eulalio A. Behm-Ansmant I. Schweizer D. Izaurralde E. (2007). P-body formation is a consequence, not the cause, of RNA-mediated gene silencing. Mol. Cell. Biol. 27 (11), 3970–3981. 10.1128/MCB.00128-07 17403906 PMC1900022

[B49] Fan A. C. Leung A. K. (2016). RNA granules and diseases: A case study of stress granules in ALS and ftld. Adv. Exp. Med. Biol. 907, 263–296. 10.1007/978-3-319-29073-7_11 27256390 PMC5247449

[B50] Fox A. H. Lamond A. I. (2010). Paraspeckles. Cold Spring Harb. Perspect. Biol. 2 (7), a000687. 10.1101/cshperspect.a000687 20573717 PMC2890200

[B51] Fox A. H. Nakagawa S. Hirose T. Bond C. S. (2018). Paraspeckles: Where long noncoding RNA meets phase separation. Trends biochem. Sci. 43 (2), 124–135. 10.1016/j.tibs.2017.12.001 29289458

[B52] Fratta P. Collins T. Pemble S. Nethisinghe S. Devoy A. Giunti P. (2014). Sequencing analysis of the spinal bulbar muscular atrophy CAG expansion reveals absence of repeat interruptions. Neurobiol. Aging 35 (2), e1–e3. 10.1016/j.neurobiolaging.2013.07.015 24041967 PMC3898077

[B53] Fritzsche R. Karra D. Bennett K. L. Ang F. Y. Heraud-Farlow J. E. Tolino M. (2013). Interactome of two diverse RNA granules links mRNA localization to translational repression in neurons. Cell Rep. 5 (6), 1749–1762. 10.1016/j.celrep.2013.11.023 24360960

[B54] Frottin F. Pérez-Berlanga M. Hartl F. U. Hipp M. S. (2021). Multiple pathways of toxicity induced by C9orf72 dipeptide repeat aggregates and G4C2 RNA in a cellular model. eLife 10, e62718. 10.7554/eLife.62718 34161229 PMC8221807

[B55] Galganski L. Urbanek M. O. Krzyzosiak W. J. (2017). Nuclear speckles: Molecular organization, biological function and role in disease. Nucleic Acids Res. 45 (18), 10350–10368. 10.1093/nar/gkx759 28977640 PMC5737799

[B56] Griesche N. Schilling J. Weber S. Rohm M. Pesch V. Matthes F. (2016). Regulation of mRNA translation by MID1: A common mechanism of expanded CAG repeat RNAs. Front. Cell. Neurosci. 10, 226. 10.3389/fncel.2016.00226 27774050 PMC5054010

[B57] Gulyurtlu S. Magon M. S. Guest P. Papavasiliou P. P. Morrison K. D. Prescott A. R. (2022). Condensation properties of stress granules and processing bodies are compromised in myotonic dystrophy type 1. Dis. Model Mech., dmm049294. 35642886 10.1242/dmm.049294 PMC9366894

[B58] Gumy L. F. Yeo G. S. Tung Y. C. Zivraj K. H. Willis D. Coppola G. (2011). Transcriptome analysis of embryonic and adult sensory axons reveals changes in mRNA repertoire localization. RNA (New York, NY) 17 (1), 85–98. 10.1261/rna.2386111 PMC3004069 21098654

[B59] Haimovich G. Ecker C. M. Dunagin M. C. Eggan E. Raj A. Gerst J. E. (2017). Intercellular mRNA trafficking via membrane nanotube-like extensions in mammalian cells. Proc. Natl. Acad. Sci. U. S. A. 114 (46), E9873–E9882. 10.1073/pnas.1706365114 29078295 PMC5699038

[B60] Hardy J. Selkoe D. J. (2002). The amyloid hypothesis of Alzheimer's disease: Progress and problems on the road to therapeutics. Sci. (New York, NY) 297 (5580), 353–356. 10.1126/science.1072994 12130773

[B61] Heinz A. Nabariya D. K. Krauss S. (2021). Huntingtin and its role in mechanisms of RNA-mediated toxicity. Toxins 13 (7), 487. 10.3390/toxins13070487 34357961 PMC8310054

[B62] Heinz A. Nabariya D. K. Krauss S. (2021). Huntington’s disease and neurodegeneration. Handbook of neurotoxicity. Cham: Springer.

[B63] Heinz A. Schilling J. van Roon-Mom W. Krauß S. (2021). The MID1 protein: A promising therapeutic target in huntington's disease. Front. Genet. 12, 761714. 10.3389/fgene.2021.761714 34659371 PMC8517220

[B64] Herrera F. Tenreiro S. Miller-Fleming L. Outeiro T. F. (2011). Visualization of cell-to-cell transmission of mutant huntingtin oligomers. PLoS Curr. 3, RRN1210. 21331289 10.1371/currents.RRN1210 PMC3037516

[B65] Hire R. R. Katrak S. M. Vaidya S. Radhakrishnan K. Seshadri M. (2011). Spinocerebellar ataxia type 17 in Indian patients: Two rare cases of homozygous expansions. Clin. Genet. 80 (5), 472–477. 10.1111/j.1399-0004.2010.01589.x 21108634

[B66] HJH (2010). The ESCRT complexes. Crit. Rev. Biochem. Mol. Biol. 45 (6), 463–487. 20653365 10.3109/10409238.2010.502516 PMC2988974

[B67] Ho T. H. Savkur R. S. Poulos M. G. Mancini M. A. Swanson M. S. Cooper T. A. (2005). Colocalization of muscleblind with RNA foci is separable from mis-regulation of alternative splicing in myotonic dystrophy. J. Cell Sci. 118 (13), 2923–2933. 10.1242/jcs.02404 15961406

[B68] Hochberg-Laufer H. Schwed-Gross A. Neugebauer K. M. Shav-Tal Y. (2019). Uncoupling of nucleo-cytoplasmic RNA export and localization during stress. Nucleic Acids Res. 47 (9), 4778–4797. 10.1093/nar/gkz168 30864659 PMC6511838

[B69] Holt C. E. Schuman E. M. (2013). The central dogma decentralized: New perspectives on RNA function and local translation in neurons. Neuron 80 (3), 648–657. 10.1016/j.neuron.2013.10.036 24183017 PMC3820025

[B70] Hong Y. Zhao T. Li X. J. Li S. (2017). Mutant huntingtin inhibits αb-crystallin expression and impairs exosome secretion from astrocytes. J. Neurosci. 37 (39), 9550–9563. 10.1523/JNEUROSCI.1418-17.2017 28893927 PMC5618269

[B71] Hou X. Gong X. Zhang L. Li T. Yuan H. Xie Y. (2019). Identification of a potential exosomal biomarker in spinocerebellar ataxia Type 3/Machado-Joseph disease. Epigenomics 11 (9), 1037–1056. 10.2217/epi-2019-0081 31144508

[B72] Hubstenberger A. Courel M. Bénard M. Souquere S. Ernoult-Lange M. Chouaib R. (2017). P-body purification reveals the condensation of repressed mRNA regulons. Mol. Cell 68 (1), 144–157. e5. 10.1016/j.molcel.2017.09.003 28965817

[B73] Huichalaf C. Sakai K. Jin B. Jones K. Wang G. L. Schoser B. (2010). Expansion of CUG RNA repeats causes stress and inhibition of translation in myotonic dystrophy 1 (DM1) cells. FASEB J. official Publ. Fed. Am. Soc. Exp. Biol. 24 (10), 3706–3719. 10.1096/fj.09-151159 PMC2996918 20479119

[B74] Hung M. E. Leonard J. N. (2016). A platform for actively loading cargo RNA to elucidate limiting steps in EV-mediated delivery. J. Extracell. Vesicles 5, 31027. 10.3402/jev.v5.31027 27189348 PMC4870355

[B75] Ikeda Y. Shizuka M. Watanabe M. Okamoto K. Shoji M. (2000). Molecular and clinical analyses of spinocerebellar ataxia type 8 in Japan. Neurology 54 (4), 950–955. 10.1212/wnl.54.4.950 10690991

[B76] Jain S. Wheeler J. R. Walters R. W. Agrawal A. Barsic A. Parker R. (2016). ATPase-modulated stress granules contain a diverse proteome and substructure. Cell 164 (3), 487–498. 10.1016/j.cell.2015.12.038 26777405 PMC4733397

[B77] Jeon I. Cicchetti F. Cisbani G. Lee S. Li E. Bae J. (2016). Human-to-mouse prion-like propagation of mutant huntingtin protein. Acta Neuropathol. 132 (4), 577–592. 10.1007/s00401-016-1582-9 27221146 PMC5023734

[B78] Jin Y. N. Johnson G. V. (2010). The interrelationship between mitochondrial dysfunction and transcriptional dysregulation in Huntington disease. J. Bioenerg. Biomembr. 42 (3), 199–205. 10.1007/s10863-010-9286-7 20556492 PMC2913874

[B79] Jønson L. Vikesaa J. Krogh A. Nielsen L. K. Hansen T. v. Borup R. (2007). Molecular composition of IMP1 ribonucleoprotein granules. Mol. Cell. Proteomics 6 (5), 798–811. 10.1074/mcp.M600346-MCP200 17289661

[B80] Kaehler C. Isensee J. Nonhoff U. Terrey M. Hucho T. Lehrach H. (2012). Ataxin-2-like is a regulator of stress granules and processing bodies. PloS one 7 (11), e50134. 10.1371/journal.pone.0050134 23209657 PMC3507954

[B81] Kalra H. Drummen G. P. Mathivanan S. (2016). Focus on extracellular vesicles: Introducing the next small big thing. Int. J. Mol. Sci. 17 (2), 170. 10.3390/ijms17020170 26861301 PMC4783904

[B82] Kanada M. Bachmann M. H. Hardy J. W. Frimannson D. O. Bronsart L. Wang A. (2015). Differential fates of biomolecules delivered to target cells via extracellular vesicles. Proc. Natl. Acad. Sci. U. S. A. 112 (12), E1433–E1442. 10.1073/pnas.1418401112 25713383 PMC4378439

[B83] Karttunen J. Heiskanen M. Navarro-Ferrandis V. Das Gupta S. Lipponen A. Puhakka N. (2018). Precipitation-based extracellular vesicle isolation from rat plasma co-precipitate vesicle-free microRNAs. J. Extracell. Vesicles 8 (1), 1555410. 10.1080/20013078.2018.1555410 30574280 PMC6300090

[B84] Kawabe Y. Mori K. Yamashita T. Gotoh S. Ikeda M. (2020e10270). The RNA exosome complex degrades expanded hexanucleotide repeat RNA in C9orf72 FTLD/ALS. EMBO J. 39, e102700. 10.15252/embj.2019102700 PMC7527818 32830871

[B85] Kawaguchi Y. Okamoto T. Taniwaki M. Aizawa M. Inoue M. Katayama S. (1994). CAG expansions in a novel gene for Machado-Joseph disease at chromosome 14q32.1. Nat. Genet. 8 (3), 221–228. 10.1038/ng1194-221 7874163

[B86] KD (2017). There is an inclusion for that: Material properties of protein granules provide a platform for building diverse cellular functions. Trends Biochem. Sci. 42 (10), 765–776. 28864230 10.1016/j.tibs.2017.08.002

[B87] Kedersha N. Anderson P. (2007). Mammalian stress granules and processing bodies. Methods Enzymol. 431, 61–81. 10.1016/S0076-6879(07)31005-7 17923231

[B88] Kedersha N. Ivanov P. Anderson P. (2013). Stress granules and cell signaling: More than just a passing phase? Trends biochem. Sci. 38 (10), 494–506. 10.1016/j.tibs.2013.07.004 24029419 PMC3832949

[B89] Kedersha N. Panas M. D. Achorn C. A. Lyons S. Tisdale S. Hickman T. (2016). G3BP-Caprin1-USP10 complexes mediate stress granule condensation and associate with 40S subunits. J. Cell Biol. 212 (7), 845–860. 10.1083/jcb.201508028 27022092 PMC4810302

[B90] Kedersha N. Stoecklin G. Ayodele M. Yacono P. Lykke-Andersen J. Fritzler M. J. (2005). Stress granules and processing bodies are dynamically linked sites of mRNP remodeling. J. Cell Biol. 169 (6), 871–884. 10.1083/jcb.200502088 15967811 PMC2171635

[B91] Keerthikumar S. Gangoda L. Liem M. Fonseka P. Atukorala I. Ozcitti C. (2015). Proteogenomic analysis reveals exosomes are more oncogenic than ectosomes. Oncotarget 6 (17), 15375–15396. 10.18632/oncotarget.3801 25944692 PMC4558158

[B92] Khandjian E. W. Tournier B. Seguin S. Tremblay S. Koninck P. D. Davidovic L. (2009). RNA granules: Functions within presynaptic terminals and postsynaptic spines: Reference module in biomedical sciences. Elsevier.

[B93] Khanna N. Hu Y. Belmont A. S. (2014). HSP70 transgene directed motion to nuclear speckles facilitates heat shock activation. Curr. Biol. 24 (10), 1138–1144. 10.1016/j.cub.2014.03.053 24794297 PMC4030642

[B94] Khong A. Matheny T. Jain S. Mitchell S. F. Wheeler J. R. Parker R. (2017). The stress granule transcriptome reveals principles of mRNA accumulation in stress granules. Mol. Cell 68 (4), 808–820. e5. 10.1016/j.molcel.2017.10.015 29129640 PMC5728175

[B95] Kiebler M. A. Bassell G. J. (2006). Neuronal RNA granules: Movers and makers. Neuron 51 (6), 685–690. 10.1016/j.neuron.2006.08.021 16982415

[B96] Kim T. H. Tsang B. Vernon R. M. Sonenberg N. Kay L. E. Forman-Kay J. D. (2019). Phospho-dependent phase separation of FMRP and CAPRIN1 recapitulates regulation of translation and deadenylation. Science 365 (6455), 825–829. 10.1126/science.aax4240 31439799

[B97] Koide R. Ikeuchi T. Onodera O. Tanaka H. Igarashi S. Endo K. (1994). Unstable expansion of CAG repeat in hereditary dentatorubral-pallidoluysian atrophy (DRPLA). Nat. Genet. 6 (1), 9–13. 10.1038/ng0194-9 8136840

[B98] Koutsoulidou A. Photiades M. Kyriakides T. C. Georgiou K. Prokopi M. Kapnisis K. (2017). Identification of exosomal muscle-specific miRNAs in serum of myotonic dystrophy patients relating to muscle disease progress. Hum. Mol. Genet. 26 (17), 3285–3302. 10.1093/hmg/ddx212 28637233

[B99] Krichevsky A. M. Kosik K. S. (2001). Neuronal RNA granules: A link between RNA localization and stimulation-dependent translation. Neuron 32 (4), 683–696. 10.1016/s0896-6273(01)00508-6 11719208

[B100] Kumar V. Hasan G. M. Hassan M. I. (2017). Unraveling the role of RNA mediated toxicity of C9orf72 repeats in C9-FTD/ALS. Front. Neurosci. 11, 711. 10.3389/fnins.2017.00711 29326544 PMC5736982

[B101] Kwon I. Xiang S. Kato M. Wu L. Theodoropoulos P. Wang T. (2014). Poly-dipeptides encoded by the C9orf72 repeats bind nucleoli, impede RNA biogenesis, and kill cells. Sci. (New York, NY) 345 (6201), 1139–1145. 10.1126/science.1254917 PMC4459787 25081482

[B102] LaCroix A. J. Stabley D. Sahraoui R. Adam M. P. Mehaffey M. Kernan K. (2019). GGC repeat expansion and exon 1 methylation of XYLT1 is a common pathogenic variant in baratela-scott syndrome. Am. J. Hum. Genet. 104 (1), 35–44. 10.1016/j.ajhg.2018.11.005 30554721 PMC6323552

[B103] Lai A. Valdez-Sinon A. N. Bassell G. J. (2020). Regulation of RNA granules by FMRP and implications for neurological diseases. Traffic (Copenhagen, Den. 21 (7), 454–462. 10.1111/tra.12733 PMC7377269 32374065

[B104] Lallemand-Breitenbach V. de Thé H. (2018). PML nuclear bodies: From architecture to function. Curr. Opin. Cell Biol. 52, 154–161. 10.1016/j.ceb.2018.03.011 29723661

[B105] Lee E. K. Kim H. H. Kuwano Y. Abdelmohsen K. Srikantan S. Subaran S. S. (2010). hnRNP C promotes APP translation by competing with FMRP for APP mRNA recruitment to P bodies. Nat. Struct. Mol. Biol. 17 (6), 732–739. 10.1038/nsmb.1815 20473314 PMC2908492

[B106] Lee H. Li C. Zhang Y. Zhang D. Otterbein L. E. Jin Y. (2019). Caveolin-1 selectively regulates microRNA sorting into microvesicles after noxious stimuli. J. Exp. Med. 216 (9), 2202–2220. 10.1084/jem.20182313 31235510 PMC6719430

[B107] Lee M. Im W. Kim M. (2021). Exosomes as a potential messenger unit during heterochronic parabiosis for amelioration of Huntington's disease. Neurobiol. Dis. 155, 105374. 10.1016/j.nbd.2021.105374 33940179

[B108] Lee M. Liu T. Im W. Kim M. (2016). Exosomes from adipose-derived stem cells ameliorate phenotype of Huntington's disease *in vitro* model. Eur. J. Neurosci. 44 (4), 2114–2119. 10.1111/ejn.13275 27177616

[B109] Lee S. T. Im W. Ban J. J. Lee M. Jung K. H. Lee S. K. (2017). Exosome-based delivery of miR-124 in a huntington's disease model. J. Mov. Disord. 10 (1), 45–52. 10.14802/jmd.16054 28122430 PMC5288667

[B110] Leidal A. M. Huang H. H. Marsh T. Solvik T. Zhang D. Ye J. (2020). The LC3-conjugation machinery specifies the loading of RNA-binding proteins into extracellular vesicles. Nat. Cell Biol. 22 (2), 187–199. 10.1038/s41556-019-0450-y 31932738 PMC7007875

[B111] Li K. Wong D. K. Hong K. Y. Raffai R. L. (2018). Cushioned-density gradient ultracentrifugation (C-dguc): A refined and high performance method for the isolation, characterization, and use of exosomes. Methods Mol. Biol. 1740, 69–83. 10.1007/978-1-4939-7652-2_7 29388137 PMC6476194

[B112] Li L. Saegusa H. Tanabe T. (2009). Deficit of heat shock transcription factor 1-heat shock 70 kDa protein 1A axis determines the cell death vulnerability in a model of spinocerebellar ataxia type 6. Genes cells. 14 (11), 1253–1269. 10.1111/j.1365-2443.2009.01348.x 19817876

[B113] Li M. Ona V. O. Guégan C. Chen M. Jackson-Lewis V. Andrews L. J. (2000). Functional role of caspase-1 and caspase-3 in an ALS transgenic mouse model. Sci. (New York, NY) 288 (5464), 335–339. 10.1126/science.288.5464.335 10764647

[B114] Liangsupree T. Multia E. Riekkola M. L. (2021). Modern isolation and separation techniques for extracellular vesicles. J. Chromatogr. A 1636, 461773. 10.1016/j.chroma.2020.461773 33316564

[B115] Liu S. Hossinger A. Heumüller S. E. Hornberger A. Buravlova O. Konstantoulea K. (2021). Highly efficient intercellular spreading of protein misfolding mediated by viral ligand-receptor interactions. Nat. Commun. 12 (1), 5739. 10.1038/s41467-021-25855-2 34667166 PMC8526834

[B116] Liu X. M. Ma L. Schekman R. (2021). Selective sorting of microRNAs into exosomes by phase-separated YBX1 condensates. eLife 10, e71982. 10.7554/eLife.71982 34766549 PMC8612733

[B117] LL (2019). Phase-to-Phase with nucleoli - stress responses, protein aggregation and novel roles of RNA. Front. Cell. Neurosci. 13, 151. 31080406 10.3389/fncel.2019.00151 PMC6497782

[B118] Loschi M. Leishman C. C. Berardone N. Boccaccio G. L. (2009). Dynein and kinesin regulate stress-granule and P-body dynamics. J. Cell Sci. 122, 3973–3982. 10.1242/jcs.051383 19825938 PMC2773196

[B119] Lötvall J. Hill A. F. Hochberg F. Buzás E. I. Di Vizio D. Gardiner C. (2014). Minimal experimental requirements for definition of extracellular vesicles and their functions: A position statement from the international society for extracellular vesicles. J. Extracell. Vesicles 3, 26913. 10.3402/jev.v3.26913 25536934 PMC4275645

[B120] Luo Y. Na Z. Slavoff S. A. (2018). P-Bodies: Composition, properties, and functions. Biochemistry 57 (17), 2424–2431. 10.1021/acs.biochem.7b01162 29381060 PMC6296482

[B121] Ma B. Savas J. N. Yu M. S. Culver B. P. Chao M. V. Tanese N. (2011). Huntingtin mediates dendritic transport of β-actin mRNA in rat neurons. Sci. Rep. 1, 140. 10.1038/srep00140 22355657 PMC3216621

[B122] Maharjan N. Künzli C. Buthey K. Saxena S. (2017). C9ORF72 regulates stress granule formation and its deficiency impairs stress granule assembly, hypersensitizing cells to stress. Mol. Neurobiol. 54 (4), 3062–3077. 10.1007/s12035-016-9850-1 27037575

[B123] Mahboubi H. Stochaj U. (2014). Nucleoli and stress granules: Connecting distant relatives. Traffic (Copenhagen, Den. 15 (10), 1179–1193. 10.1111/tra.12191 24990581

[B124] Maher-Laporte M. Berthiaume F. Moreau M. Julien L. A. Lapointe G. Mourez M. (2010). Molecular composition of staufen2-containing ribonucleoproteins in embryonic rat brain. PloS one 5 (6), e11350. 10.1371/journal.pone.0011350 20596529 PMC2893162

[B125] Maia J. Batista S. Couto N. Gregório A. C. Bodo C. Elzanowska J. (2020). Employing flow cytometry to extracellular vesicles sample microvolume analysis and quality control. Front. Cell Dev. Biol. 8, 593750. 10.3389/fcell.2020.593750 33195266 PMC7661467

[B126] Markmiller S. Soltanieh S. Server K. L. Mak R. Jin W. Fang M. Y. (2018). Context-dependent and disease-specific diversity in protein interactions within stress granules. Cell 172 (3), 590–604. e13. 10.1016/j.cell.2017.12.032 29373831 PMC5969999

[B127] Mashouri L. Yousefi H. Aref A. R. Ahadi A. M. Molaei F. Alahari S. K. (2019). Exosomes: Composition, biogenesis, and mechanisms in cancer metastasis and drug resistance. Mol. Cancer 18 (1), 75. 10.1186/s12943-019-0991-5 30940145 PMC6444571

[B128] Matheny T. Rao B. S. Parker R. (2019). Transcriptome-wide comparison of stress granules and P-bodies reveals that translation plays a major role in RNA partitioning. Mol. Cell. Biol. 39 (24), e00313–e00319. 10.1128/MCB.00313-19 31591142 PMC6879202

[B129] Mathivanan S. Ji H. Simpson R. J. (2010). Exosomes: Extracellular organelles important in intercellular communication. J. Proteomics 73 (10), 1907–1920. 10.1016/j.jprot.2010.06.006 20601276

[B130] Matsumoto J. Stewart T. Banks W. A. Zhang J. (2017). The transport mechanism of extracellular vesicles at the blood-brain barrier. Curr. Pharm. Des. 23 (40), 6206–6214. 10.2174/1381612823666170913164738 28914201

[B131] McEachin Z. T. Parameswaran J. Raj N. Bassell G. J. Jiang J. (2020). RNA-mediated toxicity in C9orf72 ALS and FTD. Neurobiol. Dis. 145, 105055. 10.1016/j.nbd.2020.105055 32829028 PMC7572710

[B132] Minciacchi V. R. You S. Spinelli C. Morley S. Zandian M. Aspuria P. J. (2015). Large oncosomes contain distinct protein cargo and represent a separate functional class of tumor-derived extracellular vesicles. Oncotarget 6 (13), 11327–11341. 10.18632/oncotarget.3598 25857301 PMC4484459

[B133] Mittelbrunn M. Gutiérrez-Vázquez C. Villarroya-Beltri C. González S. Sánchez-Cabo F. González M. A. (2011). Unidirectional transfer of microRNA-loaded exosomes from T cells to antigen-presenting cells. Nat. Commun. 2 (1), 282. 10.1038/ncomms1285 21505438 PMC3104548

[B134] Montecalvo A. Larregina A. T. Shufesky W. J. Stolz D. B. Sullivan M. L. Karlsson J. M. (2012). Mechanism of transfer of functional microRNAs between mouse dendritic cells via exosomes. Blood 119 (3), 756–766. 10.1182/blood-2011-02-338004 22031862 PMC3265200

[B135] Moujaber O. Stochaj U. (2018). Cytoplasmic RNA granules in somatic maintenance. Gerontology 64 (5), 485–494. 10.1159/000488759 29847814

[B136] Mulcahy L. A. Pink R. C. Carter D. R. (2014). Routes and mechanisms of extracellular vesicle uptake. J. Extracell. Vesicles 3, 24641. 10.3402/jev.v3.24641 PMC4122821 25143819

[B137] Musova Z. Mazanec R. Krepelova A. Ehler E. Vales J. Jaklova R. (2009). Highly unstable sequence interruptions of the CTG repeat in the myotonic dystrophy gene. Am. J. Med. Genet. A 149A (7), 1365–1374. 10.1002/ajmg.a.32987 19514047

[B220] Myers R. H. (2004). Huntington’s disease genetics. NeuroRx 1 (2), 255–262. 10.1602/neurorx.1.2.255 15717026 PMC534940

[B138] Nabariya D. K. Pallu R. Yenuganti V. R. (2020). Exosomes: The protagonists in the tale of colorectal cancer? Biochim. Biophys. Acta. Rev. Cancer 1874 (2), 188426. 10.1016/j.bbcan.2020.188426 32956762

[B139] Nalavade R. Griesche N. Ryan D. P. Hildebrand S. Krauss S. (2013). Mechanisms of RNA-induced toxicity in CAG repeat disorders. Cell Death Dis. 4, e752. 10.1038/cddis.2013.276 23907466 PMC3763438

[B140] Nalavadi V. C. Muddashetty R. S. Gross C. Bassell G. J. (2012). Dephosphorylation-induced ubiquitination and degradation of FMRP in dendrites: A role in immediate early mGluR-stimulated translation. J. Neurosci. 32 (8), 2582–2587. 10.1523/JNEUROSCI.5057-11.2012 22357842 PMC3427762

[B141] Namkoong S. Ho A. Woo Y. M. Kwak H. Lee J. H. (2018). Systematic characterization of stress-induced RNA granulation. Mol. Cell 70 (1), 175–187. e8. 10.1016/j.molcel.2018.02.025 29576526 PMC6359928

[B142] Niss F. Pinero-Paez L. Zaidi W. Hallberg E. Strom A. L. (2022). Key modulators of the stress granule response TIA1, TDP-43, and G3BP1 are altered by polyglutamine-expanded ATXN7. Mol. Neurobiol. 59, 5236–5251. 10.1007/s12035-022-02888-2 35689166 PMC9363381

[B143] O'Hearn E. Holmes S. E. Margolis R. L. (2012). Spinocerebellar ataxia type 12. Handb. Clin. Neurol. 103, 535–547. 10.1016/B978-0-444-51892-7.00034-6 21827912

[B144] Onomoto K. Jogi M. Yoo J. S. Narita R. Morimoto S. Takemura A. (2012). Critical role of an antiviral stress granule containing RIG-I and PKR in viral detection and innate immunity. PloS one 7 (8), e43031. 10.1371/journal.pone.0043031 22912779 PMC3418241

[B145] Orr H. T. Zoghbi H. Y. (2007). Trinucleotide repeat disorders. Annu. Rev. Neurosci. 30, 575–621. 10.1146/annurev.neuro.29.051605.113042 17417937

[B146] Palicharla V. R. Maddika S. (2015). HACE1 mediated K27 ubiquitin linkage leads to YB-1 protein secretion. Cell. Signal. 27 (12), 2355–2362. 10.1016/j.cellsig.2015.09.001 26343856

[B147] Parker R. Sheth U. (2007). P bodies and the control of mRNA translation and degradation. Mol. Cell 25 (5), 635–646. 10.1016/j.molcel.2007.02.011 17349952

[B148] Parolini I. Federici C. Raggi C. Lugini L. Palleschi S. De Milito A. (2009). Microenvironmental pH is a key factor for exosome traffic in tumor cells. J. Biol. Chem. 284 (49), 34211–34222. 10.1074/jbc.M109.041152 19801663 PMC2797191

[B149] Paul S. Dansithong W. Jog S. P. Holt I. Mittal S. Brook J. D. (2011). Expanded CUG repeats Dysregulate RNA splicing by altering the stoichiometry of the muscleblind 1 complex. J. Biol. Chem. 286, 38427–38438. 10.1074/jbc.M111.255224 21900255 PMC3207417

[B150] Pecho-Vrieseling E. Rieker C. Fuchs S. Bleckmann D. Esposito M. S. Botta P. (2014). Transneuronal propagation of mutant huntingtin contributes to non-cell autonomous pathology in neurons. Nat. Neurosci. 17 (8), 1064–1072. 10.1038/nn.3761 25017010

[B151] Peters L. Meister G. (2007). Argonaute proteins: Mediators of RNA silencing. Mol. Cell 26 (5), 611–623. 10.1016/j.molcel.2007.05.001 17560368

[B152] Pircs K. Petri R. Madsen S. Brattås P. L. Vuono R. Ottosson D. R. (2018). Huntingtin aggregation impairs autophagy, leading to argonaute-2 accumulation and global MicroRNA dysregulation. Cell Rep. 24 (6), 1397–1406. 10.1016/j.celrep.2018.07.017 30089251

[B153] Poteryaev D. Datta S. Ackema K. Zerial M. Spang A. (2010). Identification of the switch in early-to-late endosome transition. Cell 141 (3), 497–508. 10.1016/j.cell.2010.03.011 20434987

[B154] Povea-Cabello S. Oropesa-Ávila M. de la Cruz-Ojeda P. Villanueva-Paz M. de la Mata M. Suárez-Rivero J. M. (2017). Dynamic reorganization of the cytoskeleton during apoptosis: The two coffins hypothesis. Int. J. Mol. Sci. 18 (11), 2393. 10.3390/ijms18112393 29137119 PMC5713361

[B155] Protter D. Parker R. (2016). Principles and properties of stress granules. Trends Cell Biol. 26 (9), 668–679. 10.1016/j.tcb.2016.05.004 27289443 PMC4993645

[B156] Pushpalatha K. V. Besse F. (2019). Local translation in axons: When membraneless RNP granules meet membrane-bound organelles. Front. Mol. Biosci. 6, 129. 10.3389/fmolb.2019.00129 31824961 PMC6882739

[B157] Ratajczak J. Miekus K. Kucia M. Zhang J. Reca R. Dvorak P. (2006). Embryonic stem cell-derived microvesicles reprogram hematopoietic progenitors: Evidence for horizontal transfer of mRNA and protein delivery. Leukemia 20 (5), 847–856. 10.1038/sj.leu.2404132 16453000

[B158] Ravel-Chapuis A. Klein Gunnewiek A. Bélanger G. Crawford Parks T. E. Côté J. Jasmin B. J. (2016). Staufen1 impairs stress granule formation in skeletal muscle cells from myotonic dystrophy type 1 patients. Mol. Biol. Cell 27 (11), 1728–1739. 10.1091/mbc.E15-06-0356 27030674 PMC4884064

[B159] Reineke L. C. Kedersha N. Langereis M. A. van Kuppeveld F. J. Lloyd R. E. (2015). Stress granules regulate double-stranded RNA-dependent protein kinase activation through a complex containing G3BP1 and Caprin1. mBio 6 (2), e02486. 10.1128/mBio.02486-14 25784705 PMC4453520

[B160] Reineke L. C. Lloyd R. E. (2015). The stress granule protein G3BP1 recruits protein kinase R to promote multiple innate immune antiviral responses. J. Virol. 89 (5), 2575–2589. 10.1128/JVI.02791-14 25520508 PMC4325707

[B161] Ren P. H. Lauckner J. E. Kachirskaia I. Heuser J. E. Melki R. Kopito R. R. (2009). Cytoplasmic penetration and persistent infection of mammalian cells by polyglutamine aggregates. Nat. Cell Biol. 11 (2), 219–225. 10.1038/ncb1830 19151706 PMC2757079

[B162] Richter J. D. Zhao X. (2021). The molecular biology of FMRP: New insights into fragile X syndrome. Nat. Rev. Neurosci. 22 (4), 209–222. 10.1038/s41583-021-00432-0 33608673 PMC8094212

[B163] Rossi S. Rompietti V. Antonucci Y. Giovannini D. Scopa C. Scaricamazza S. (2020). UsnRNP trafficking is regulated by stress granules and compromised by mutant ALS proteins. Neurobiol. Dis. 138, 104792. 10.1016/j.nbd.2020.104792 32027933

[B164] Russo I. Bubacco L. Greggio E. (2012). Exosomes-associated neurodegeneration and progression of Parkinson's disease. Am. J. Neurodegener. Dis. 1 (3), 217–225. 23383394 PMC3560468

[B165] Sanchez II Nguyen T. B. England W. E. Lim R. G. Vu A. Q. Miramontes R. (2021). Huntington's disease mice and human brain tissue exhibit increased G3BP1 granules and TDP43 mislocalization. J. Clin. Invest. 131 (12), e140723. 10.1172/JCI140723 33945510 PMC8203471

[B166] Sathasivam K. Neueder A. Gipson T. A. Landles C. Benjamin A. C. Bondulich M. K. (2013). Aberrant splicing of HTT generates the pathogenic exon 1 protein in Huntington disease. Proc. Natl. Acad. Sci. U. S. A. 110 (6), 2366–2370. 10.1073/pnas.1221891110 23341618 PMC3568346

[B167] Savas J. N. Ma B. Deinhardt K. Culver B. P. Restituito S. Wu L. (2010). A role for huntington disease protein in dendritic RNA granules. J. Biol. Chem. 285 (17), 13142–13153. 10.1074/jbc.M110.114561 20185826 PMC2857123

[B168] Savas J. N. Makusky A. Ottosen S. Baillat D. Then F. Krainc D. (2008). Huntington's disease protein contributes to RNA-mediated gene silencing through association with Argonaute and P bodies. Proc. Natl. Acad. Sci. U. S. A. 105 (31), 10820–10825. 10.1073/pnas.0800658105 18669659 PMC2504805

[B169] Schilling J. Broemer M. Atanassov I. Duernberger Y. Vorberg I. Dieterich C. (2019). Deregulated splicing is a major mechanism of RNA-induced toxicity in huntington's disease. J. Mol. Biol. 431 (9), 1869–1877. 10.1016/j.jmb.2019.01.034 30711541

[B170] Schoenberg D. R. Maquat L. E. (2012). Regulation of cytoplasmic mRNA decay. Nat. Rev. Genet. 13 (4), 246–259. 10.1038/nrg3160 22392217 PMC3351101

[B171] SDL (2006). SnapShot: Cellular bodies. Cell 127 (5), 1071. 10.1016/j.cell.2006.11.026 17129789

[B172] Sharma S. Gimzewski J. (2012). The quest for characterizing exosomes: Circulating nano-sized vesicles. J. Nanomedic. Nanotechnol. 3 (7), 1. 10.4172/2157-7439.1000e115

[B173] Shigeoka T. Jung H. Jung J. Turner-Bridger B. Ohk J. Lin J. Q. (2016). Dynamic axonal translation in developing and mature visual circuits. Cell 166 (1), 181–192. 10.1016/j.cell.2016.05.029 27321671 PMC4930487

[B174] Shurtleff M. J. Yao J. Qin Y. Nottingham R. M. Temoche-Diaz M. M. Schekman R. (2017). Broad role for YBX1 in defining the small noncoding RNA composition of exosomes. Proc. Natl. Acad. Sci. U. S. A. 114 (43), E8987–E8995. 10.1073/pnas.1712108114 29073095 PMC5663387

[B175] Solomon S. Xu Y. Wang B. David M. D. Schubert P. Kennedy D. (2007). Distinct structural features of caprin-1 mediate its interaction with G3BP-1 and its induction of phosphorylation of eukaryotic translation initiation factor 2alpha, entry to cytoplasmic stress granules, and selective interaction with a subset of mRNAs. Mol. Cell. Biol. 27 (6), 2324–2342. 10.1128/MCB.02300-06 17210633 PMC1820512

[B176] Soria Lopez J. A. González H. M. Léger G. C. (2019). Alzheimer's disease. Handb. Clin. Neurol. 167, 231–255. 10.1016/B978-0-12-804766-8.00013-3 31753135

[B177] Souquere S. Mollet S. Kress M. Dautry F. Pierron G. Weil D. (2009). Unravelling the ultrastructure of stress granules and associated P-bodies in human cells. J. Cell Sci. 122, 3619–3626. 10.1242/jcs.054437 19812307

[B178] Starke E. L. Zius K. Barbee S. A. (2022). FXS causing missense mutations disrupt FMRP granule formation, dynamics, and function. PLoS Genet. 18 (2), e1010084. 10.1371/journal.pgen.1010084 35202393 PMC8903291

[B179] Stevanin G. Giunti P. Belal G. D. Dürr A. Ruberg M. Wood N. (1998). De novo expansion of intermediate alleles in spinocerebellar ataxia 7. Hum. Mol. Genet. 7 (11), 1809–1813. 10.1093/hmg/7.11.1809 9736784

[B180] Stuffers S. Sem Wegner C. Stenmark H. Brech A. (2009). Multivesicular endosome biogenesis in the absence of ESCRTs. Traffic (Copenhagen, Den. 10 (7), 925–937. 10.1111/j.1600-0854.2009.00920.x 19490536

[B181] Syed F. Maier B. F. Anderson-Baucum E. Mastracci T. L. Evans-Molina C. Mirmira R. (2020). 82-OR: Exosomes from human ß-cells incorporate stress granule components and may serve as biomarkers of ß-cell stress in type 1 diabetes. Diabetes 69, 1. 10.2337/db20-82-or 31862689

[B182] Takahara T. Maeda T. (2012). Transient sequestration of TORC1 into stress granules during heat stress. Mol. Cell 47 (2), 242–252. 10.1016/j.molcel.2012.05.019 22727621

[B183] Takahashi M. Ishikawa K. Sato N. Obayashi M. Niimi Y. Ishiguro T. (2012). Reduced brain-derived neurotrophic factor (BDNF) mRNA expression and presence of BDNF-immunoreactive granules in the spinocerebellar ataxia type 6 (SCA6) cerebellum. Neuropathology 32 (6), 595–603. 10.1111/j.1440-1789.2012.01302.x 22393909

[B184] Taneja K. L. McCurrach M. Schalling M. Housman D. Singer R. H. (1995). Foci of trinucleotide repeat transcripts in nuclei of myotonic dystrophy cells and tissues. J. Cell Biol. 128 (6), 995–1002. 10.1083/jcb.128.6.995 7896884 PMC2120416

[B185] Tao Z. Wang H. Xia Q. Li K. Li K. Jiang X. (2015). Nucleolar stress and impaired stress granule formation contribute to C9orf72 RAN translation-induced cytotoxicity. Hum. Mol. Genet. 24 (9), 2426–2441. 10.1093/hmg/ddv005 25575510

[B186] Tenekeci U. Poppe M. Beuerlein K. Buro C. Müller H. Weiser H. (2016). K63-Ubiquitylation and TRAF6 pathways regulate mammalian P-body formation and mRNA decapping. Mol. Cell 62 (6), 540–557. 10.1016/j.molcel.2016.07.009 27315556

[B187] Théry C. Witwer K. W. Aikawa E. Alcaraz M. J. Anderson J. D. Andriantsitohaina R. (2018). Minimal information for studies of extracellular vesicles 2018 (MISEV2018): A position statement of the international society for extracellular vesicles and update of the MISEV2014 guidelines. J. Extracell. Vesicles 7 (1), 1535750. 10.1080/20013078.2018.1535750 30637094 PMC6322352

[B188] Théry C. Zitvogel L. Amigorena S. (2002). Exosomes: Composition, biogenesis and function. Nat. Rev. Immunol. 2 (8), 569–579. 10.1038/nri855 12154376

[B189] Thomas M. G. Martinez Tosar L. J. Desbats M. A. Leishman C. C. Boccaccio G. L. (2009). Mammalian Staufen 1 is recruited to stress granules and impairs their assembly. J. Cell Sci. 122, 563–573. 10.1242/jcs.038208 19193871 PMC2714435

[B190] Thornton R. J. O. C. A. (2006). RNA-dominant diseases. Human molecular genetics. Hum. Mol. Genet. 15, R162–R9. 16987879 10.1093/hmg/ddl181

[B191] Tian S. Curnutte H. A. Trcek T. (2020). RNA granules: A view from the RNA perspective. Mol. (Basel, Switz. 25 (14), 3130. 10.3390/molecules25143130 PMC7397151 32650583

[B192] Todd P. K. Paulson H. L. (2010). RNA-mediated neurodegeneration in repeat expansion disorders. Ann. Neurol. 67 (3), 291–300. 10.1002/ana.21948 20373340 PMC2852186

[B193] Tourrière H. Chebli K. Zekri L. Courselaud B. Blanchard J. M. Bertrand E. (2003). The RasGAP-associated endoribonuclease G3BP assembles stress granules. J. Cell Biol. 160 (6), 823–831. 10.1083/jcb.200212128 12642610 PMC2173781

[B194] Tricarico C. Clancy J. D'Souza-Schorey C. (2017). Biology and biogenesis of shed microvesicles. Small GTPases 8 (4), 220–232. 10.1080/21541248.2016.1215283 27494381 PMC5680703

[B195] Tripathi V. Ellis J. D. Shen Z. Song D. Y. Pan Q. Watt A. T. (2010). The nuclear-retained noncoding RNA MALAT1 regulates alternative splicing by modulating SR splicing factor phosphorylation. Mol. Cell 39 (6), 925–938. 10.1016/j.molcel.2010.08.011 20797886 PMC4158944

[B196] Tsekrekou M. Stratigi K. Chatzinikolaou G. (2017). The nucleolus: In genome maintenance and repair. Int. J. Mol. Sci. 18 (7), 1411. 10.3390/ijms18071411 28671574 PMC5535903

[B197] Urbanska A. S. Janusz-Kaminska A. Switon K. Hawthorne A. L. Perycz M. Urbanska M. (2017). ZBP1 phosphorylation at serine 181 regulates its dendritic transport and the development of dendritic trees of hippocampal neurons. Sci. Rep. 7 (1), 1876. 10.1038/s41598-017-01963-2 28500298 PMC5431813

[B198] Valadi H. Ekström K. Bossios A. Sjöstrand M. Lee J. J. Lötvall J. O. (2007). Exosome-mediated transfer of mRNAs and microRNAs is a novel mechanism of genetic exchange between cells. Nat. Cell Biol. 9 (6), 654–659. 10.1038/ncb1596 17486113

[B199] Vergauwen G. Dhondt B. Van Deun J. De Smedt E. Berx G. Timmerman E. (2017). Confounding factors of ultrafiltration and protein analysis in extracellular vesicle research. Sci. Rep. 7 (1), 2704. 10.1038/s41598-017-02599-y 28577337 PMC5457435

[B200] Wang J. Langfelder P. Horvath S. Palazzolo M. J. (2017). Exosomes and homeostatic synaptic plasticity are linked to each other and to huntington's, Parkinson's, and other neurodegenerative diseases by database-enabled analyses of comprehensively curated datasets. Front. Neurosci. 11, 149. 10.3389/fnins.2017.00149 28611571 PMC5374209

[B201] WCJ (2019). Fragile X and APP: A decade in review, a vision for the future. Mol. Neurobiol. 56 (6), 3904–3921. 30225775 10.1007/s12035-018-1344-x PMC6421119

[B202] Wen X. Tan W. Westergard T. Krishnamurthy K. Markandaiah S. S. Shi Y. (2014). Antisense proline-arginine RAN dipeptides linked to C9ORF72-ALS/FTD form toxic nuclear aggregates that initiate *in vitro* and *in vivo* neuronal death. Neuron 84 (6), 1213–1225. 10.1016/j.neuron.2014.12.010 25521377 PMC4632245

[B203] Westergard T. Jensen B. K. Wen X. Cai J. Kropf E. Iacovitti L. (2016). Cell-to-Cell transmission of dipeptide repeat proteins linked to C9orf72-ALS/FTD. Cell Rep. 17, 645–652. 10.1016/j.celrep.2016.09.032 27732842 PMC5078984

[B204] Wheeler J. R. Jain S. Khong A. Parker R. (2017). Isolation of yeast and mammalian stress granule cores. Methods (San Diego, Calif. 126, 12–17. 10.1016/j.ymeth.2017.04.020 28457979 PMC5924690

[B205] White J. P. Cardenas A. M. Marissen W. E. Lloyd R. E. (2007). Inhibition of cytoplasmic mRNA stress granule formation by a viral proteinase. Cell Host Microbe 2 (5), 295–305. 10.1016/j.chom.2007.08.006 18005751

[B206] Willemsen R. Levenga J. Oostra B. A. (2011). CGG repeat in the FMR1 gene: Size matters. Clin. Genet. 80 (3), 214–225. 10.1111/j.1399-0004.2011.01723.x 21651511 PMC3151325

[B207] Wolozin B. Ivanov P. (2019). Stress granules and neurodegeneration. Nat. Rev. Neurosci. 20 (11), 649–666. 10.1038/s41583-019-0222-5 31582840 PMC6986315

[B208] Wozniak A. L. Adams A. King K. E. Dunn W. Christenson L. K. Hung W. T. (2020). The RNA binding protein FMR1 controls selective exosomal miRNA cargo loading during inflammation. J. Cell Biol. 219 (10), e201912074. 10.1083/jcb.201912074 32970791 PMC7659717

[B209] Yang W. Dunlap J. R. Andrews R. B. Wetzel R. (2002). Aggregated polyglutamine peptides delivered to nuclei are toxic to mammalian cells. Hum. Mol. Genet. 11 (23), 2905–2917. 10.1093/hmg/11.23.2905 12393802

[B210] Yang X. Shen Y. Garre E. Hao X. Krumlinde D. Cvijović M. (2014). Stress granule-defective mutants deregulate stress responsive transcripts. PLoS Genet. 10 (11), e1004763. 10.1371/journal.pgen.1004763 25375155 PMC4222700

[B211] Yenuganti V. R. Afroz S. Khan R. A. Bharadwaj C. Nabariya D. K. Nayak N. (2022). Milk exosomes elicit a potent anti-viral activity against dengue virus. J. Nanobiotechnology 20 (1), 317. 10.1186/s12951-022-01496-5 35794557 PMC9258094

[B212] Yokoyama T. Ishiyama M. Hasegawa K. Uchihara T. Yagishita S. (2014). Novel neuronal cytoplasmic inclusions in a patient carrying SCA8 expansion mutation. Neuropathology 34 (1), 27–31. 10.1111/neup.12042 23711133

[B213] Yoon J. H. Choi E. J. Parker R. (2010). Dcp2 phosphorylation by Ste20 modulates stress granule assembly and mRNA decay in *Saccharomyces cerevisiae* . J. Cell Biol. 189 (5), 813–827. 10.1083/jcb.200912019 20513766 PMC2878948

[B214] You H. J. Fang S. B. Wu T. T. Zhang H. Feng Y. K. Li X. J. (2020). Mesenchymal stem cell-derived exosomes improve motor function and attenuate neuropathology in a mouse model of Machado-Joseph disease. Stem Cell Res. Ther. 11 (1), 222. 10.1186/s13287-020-01727-2 32513306 PMC7278177

[B215] Younas N. Fernandez Flores L. C. Hopfner F. Höglinger G. U. Zerr I. (2022). A new paradigm for diagnosis of neurodegenerative diseases: Peripheral exosomes of brain origin. Transl. Neurodegener. 11 (1), 28. 10.1186/s40035-022-00301-5 35527262 PMC9082915

[B216] Zeitelhofer M. Karra D. Macchi P. Tolino M. Thomas S. Schwarz M. (2008). Dynamic interaction between P-bodies and transport ribonucleoprotein particles in dendrites of mature hippocampal neurons. J. Neurosci. 28 (30), 7555–7562. 10.1523/JNEUROSCI.0104-08.2008 18650333 PMC6670838

[B217] Zhang N. Ashizawa T. (2017). RNA toxicity and foci formation in microsatellite expansion diseases. Curr. Opin. Genet. Dev. 44, 17–29. 10.1016/j.gde.2017.01.005 28208060 PMC5447490

[B218] Zhang X. Abels E. R. Redzic J. S. Margulis J. Finkbeiner S. Breakefield X. O. (2016). Potential transfer of polyglutamine and CAG-repeat RNA in extracellular vesicles in huntington's disease: Background and evaluation in cell culture. Cell. Mol. Neurobiol. 36 (3), 459–470. 10.1007/s10571-016-0350-7 26951563 PMC5844350

[B219] Zhang Y. Liu Y. Liu H. Tang W. H. (2019). Exosomes: Biogenesis, biologic function and clinical potential. Cell Biosci. 9 (1), 19. 10.1186/s13578-019-0282-2 30815248 PMC6377728

